# Topographic variability of the normal circle of Willis anatomy on a paediatric population

**DOI:** 10.1093/braincomms/fcab055

**Published:** 2021-04-03

**Authors:** Wael Zrafi, Cristina Veres, Volodia Dangouloff-Ros, Nathalie Boddaert, Nadia Haddy, Neige Journy, Rodrigue Allodji, Mohamad Mohamad Alabdoaburas, Ibrahima Diallo, Florent de Vathaire

**Affiliations:** Radiation Epidemiology Group, Center for Research in Epidemiology and Population Health, Institut national de la santé et de la recherche médicale (INSERM) U1018, Villejuif F-94805, France; Gustave Roussy, Villejuif F-94805, France; University Paris Saclay, Villejuif F-94805, France; Radiation Epidemiology Group, Center for Research in Epidemiology and Population Health, Institut national de la santé et de la recherche médicale (INSERM) U1018, Villejuif F-94805, France; Gustave Roussy, Villejuif F-94805, France; University Paris Saclay, Villejuif F-94805, France; Molecular Radiotherapy and Innovative Therapeutics, Institut national de la santé et de la recherche médicale (INSERM) UMR 1030, Villejuif F-94805, France; Department of Radiation Oncology, Gustave Roussy Cancer Campus, Villejuif 94805, France; Pediatric Radiology, AP-HP, Hôpital Necker Enfants Malades, Paris F-75015, France; Université de Paris, Paris F-75015, France; Institut Imagine UMR 1163, Paris F-75015, France; Institut national de la santé et de la recherche médicale (INSERM) Unit UA 10, Paris F-75015, France; Pediatric Radiology, AP-HP, Hôpital Necker Enfants Malades, Paris F-75015, France; Université de Paris, Paris F-75015, France; Institut Imagine UMR 1163, Paris F-75015, France; Institut national de la santé et de la recherche médicale (INSERM) Unit UA 10, Paris F-75015, France; Radiation Epidemiology Group, Center for Research in Epidemiology and Population Health, Institut national de la santé et de la recherche médicale (INSERM) U1018, Villejuif F-94805, France; Gustave Roussy, Villejuif F-94805, France; University Paris Saclay, Villejuif F-94805, France; Radiation Epidemiology Group, Center for Research in Epidemiology and Population Health, Institut national de la santé et de la recherche médicale (INSERM) U1018, Villejuif F-94805, France; Gustave Roussy, Villejuif F-94805, France; University Paris Saclay, Villejuif F-94805, France; Radiation Epidemiology Group, Center for Research in Epidemiology and Population Health, Institut national de la santé et de la recherche médicale (INSERM) U1018, Villejuif F-94805, France; Gustave Roussy, Villejuif F-94805, France; University Paris Saclay, Villejuif F-94805, France; Radiation Epidemiology Group, Center for Research in Epidemiology and Population Health, Institut national de la santé et de la recherche médicale (INSERM) U1018, Villejuif F-94805, France; Gustave Roussy, Villejuif F-94805, France; University Paris Saclay, Villejuif F-94805, France; Radiation Epidemiology Group, Center for Research in Epidemiology and Population Health, Institut national de la santé et de la recherche médicale (INSERM) U1018, Villejuif F-94805, France; Gustave Roussy, Villejuif F-94805, France; University Paris Saclay, Villejuif F-94805, France; Molecular Radiotherapy and Innovative Therapeutics, Institut national de la santé et de la recherche médicale (INSERM) UMR 1030, Villejuif F-94805, France; Department of Radiation Oncology, Gustave Roussy Cancer Campus, Villejuif 94805, France; Radiation Epidemiology Group, Center for Research in Epidemiology and Population Health, Institut national de la santé et de la recherche médicale (INSERM) U1018, Villejuif F-94805, France; Gustave Roussy, Villejuif F-94805, France; University Paris Saclay, Villejuif F-94805, France

**Keywords:** circle of Willis, population variability, dosimetry, radiation therapy

## Abstract

Long-term sequelae are major limitations of radiation therapy use, especially for childhood brain tumour. Circle of Willis irradiation strongly increases the long-term risk of stroke, but to establish dose-response relationship, anticipating long-term effects of new techniques, requires to perform accurate and reproducible dosimetric estimations in large cohorts of patients having received radiotherapy decades ago. For the accuracy of retrospective dose reconstruction, the topographic variability of the Circle of Willis arteries is crucial. In order to improve retrospective dosimetric studies and dose-volume estimates to the typical Circle of Willis arteries, we aim to study the inter-individual topographic variability of these structures. Thirty-eight time of flight MRI sequences of children aged 2–17 years in both genders were investigated. A region growth algorithm was used for the segmentation of the cerebral arteries. A rigid registration in a common skull was performed following the anatomy of skull base foramina. The Posterior clinoid processes of the sella turcica were used as reference landmark (R0), and 5 key landmarks were chosen in each segmented Circle of Willis, then distances between the 5 landmarks and R0 were calculated for each of the 38 subjects. The distance between R0 and each landmark of the Circle of Willis followed a normal distribution, the average values ranging from 13.6 to 17.0 mm, and the standard deviations ranged from 2.6 to 3.0 mm, i.e. less than a fifth of the average value. The perimeter of the Circle of Willis was longer in older subjects, this increase being isotropic. Our study shows a remarkably low topographic variability of the typical Circle of Willis. An important result, allowing reliable anthropomorphic phantoms-based retrospective estimations of the radiation doses delivered to these arterial structures during radiotherapy treatment.

## Teaser

Zrafi et al. report inter-individual topographic variability in the Circle of Willis structure in a paediatric population. Their work shows a remarkably low variability of the typical Circle of Willis and provide data that have potential benefit for retrospective dosimetric studies in children developing cerebrovascular diseases after radiotherapy.

## Graphical Abstract

**Graphical Abstract:**
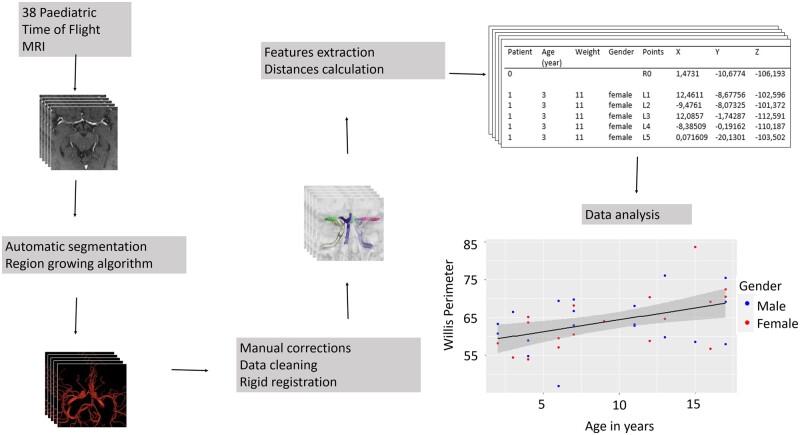

## Introduction

The 5-year survival of childhood cancer patients had been significantly improved exceeding 80% nowadays in developed European countries,^1^ due to multidisciplinary treatments, compliance with evidence-based therapeutic protocols, new drugs and technical progress of radiotherapy allowing a better targeting of tumours and sparing of healthy tissues.^2^

About 75% of paediatric cancer survivors develop at least one chronic disease during long-term follow-up such as cardiovascular, endocrine and psychological pathologies.^3–9^

Several studies have described the long-term iatrogenic effect of radiotherapy on arterial structures, mainly to the heart arteries, but also to the main brain arteries, showing a significant increase in morbidity and mortality.^4^^,^^6^^,^^8–12^

Haddy et al.^13^ had investigated 23 deaths due to cerebrovascular diseases among 4227 childhood cancer survivors, showing a 22% increase in cerebrovascular mortality for each Gray received in the prepontine cistern.

Similarly, El-Fayech et al.^14^ study showed an 8.5-fold increase in stroke risk for patients who received radiation therapy. In this study, the dose received by the Circle of Willis appears to be the best predictor of stroke risk.

Despite, the rising awareness about the radiation-related toxicity, recommendation for the contouring and dose constraints to the Circle of Willis are yet lacking.

Our aim was to investigate the topographic variability in typical Circle of Willis structures in a paediatric population and provide data that have potential benefit for retrospective dosimetric studies in children developing cerebrovascular diseases after radiotherapy.

## Methods

### Study population

We have investigated 38 consecutive time of flight MRI sequences of children aged from 2 to 17 years with no anomalies nor anatomic variant of the Circle of Willis, as confirmed by a senior paediatric neuroradiologist (VDR, 9 years of experience). There were 19 boys and 19 girls, with a median age of 8 years ranging from 2 to 17 (Supplementary Table 1). Most of them were consulting for headaches in context of sickle cell diseases, without evidence of vascular abnormality on MRI.

All MRI were performed in same Radiology department, two machine were used the DISCOVERY MR750 and the Optima MR450w, 3 and 1.5 Tesla, The slice thickness was 1.2 mm, and the reconstruction matrix size was 512 × 512 pixels for most of them.

### Image processing

Brain artery segmentation was performed using a region growing algorithm (Osirix^®^ software). The segmentation was focused on the circle of Willis, as being reported to be the main predictor of stroke risk (Supplementary Fig. 1).^3^

ISOgray™ (Dosisoft, Cachan, France) software was used to import the sequences obtained by OsiriX, and manually label each of the Circle of Willis arteries.

In order to ensure the reproducibility of the labelling of the contoured arterial structures, we follow the following labelling process:

- the left and right internal carotid arteries (ICA), each one passes through its respective carotid canal, and
- the basilar artery passes through the Magnum Foramen close to the anterior surface of the brain stem.
- These structures were segmented until the bifurcation of each one of them:
  - bifurcation of the basilar artery into left and right posterior cerebral arteries, and
  - bifurcation of each ICA into left and right middle cerebral arteries, left and right anterior cerebral arteries.
- These arteries were segmented until their first bifurcation, in order to avoid segmentation of the distal arteries.
- The communicating arteries were segmented as following:
  - the anterior communicating artery, as being median arterial segment that connects the left and right anterior cerebral arteries, and
  - the left and right posterior communicating arteries, which connects respectively each ICA to the corresponding posterior cerebral arteries

Thus, 12 structures were segmented for each MRI (38 MRIs).

**Figure 1:**
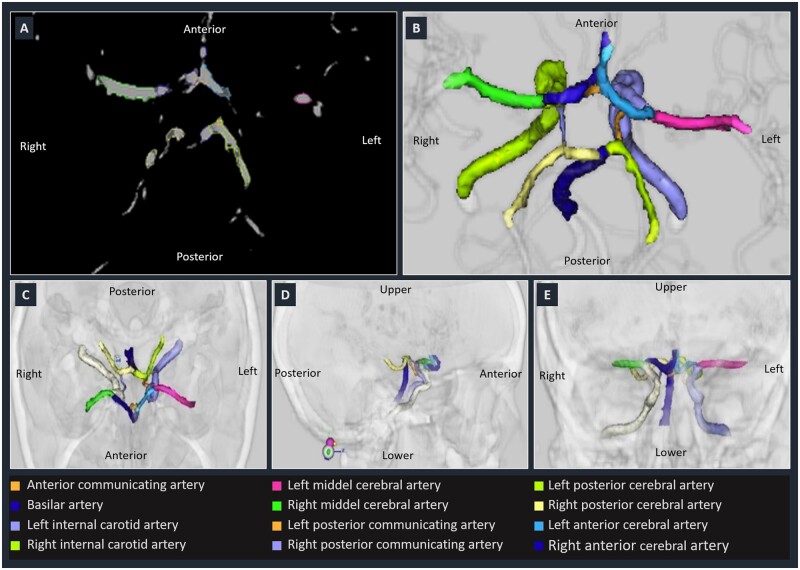
**Circle of Willis contouring, labelling and registration steps.** (**A**) Axial view of automatic segmentation with region growing algorithm. (**B**) Three-dimensional reconstruction of the Circle of Willis constituent arteries after data cleaning. (**C**–**E**) Axial sagittal and coronal views after rigid registration on a common skull.

Figure 1 shows an example of some steps and the result obtained with the above segmentation process.

### Registration

In order to investigate the topographic variability between the segmented Circle of Willis of 38 subjects, these volumes were rigidly registered in the same 3D space and a common coordinates system. We proceeded by a matching the segmented Circle of Willis to a skull bone imaging respecting the different anatomical landmarks, in particular the base of skull foramina of the left and right ICA in the skull base.

This technique allows to adjust and take into account the rotational and translational differences in position of the head when performing the MRI, and to obtain a good registration of the different Circle of Willis.

### Variability investigations

To study the topographic variability of the Circle of Willis, we defined five easily identifiable anatomical landmarks in each one.

L1: Bifurcation of the right ICA in right anterior cerebral arteries and middle cerebral arteries.

L2: Bifurcation of the left ICA in left anterior cerebral arteries and middle cerebral arteries.

L3: Top of the right cavernous sinus.

L4: Top of the left cavernous sinus.

L5: Bifurcation of the basilar artery in left and right posterior cerebral arteries.

**Figure 2:**
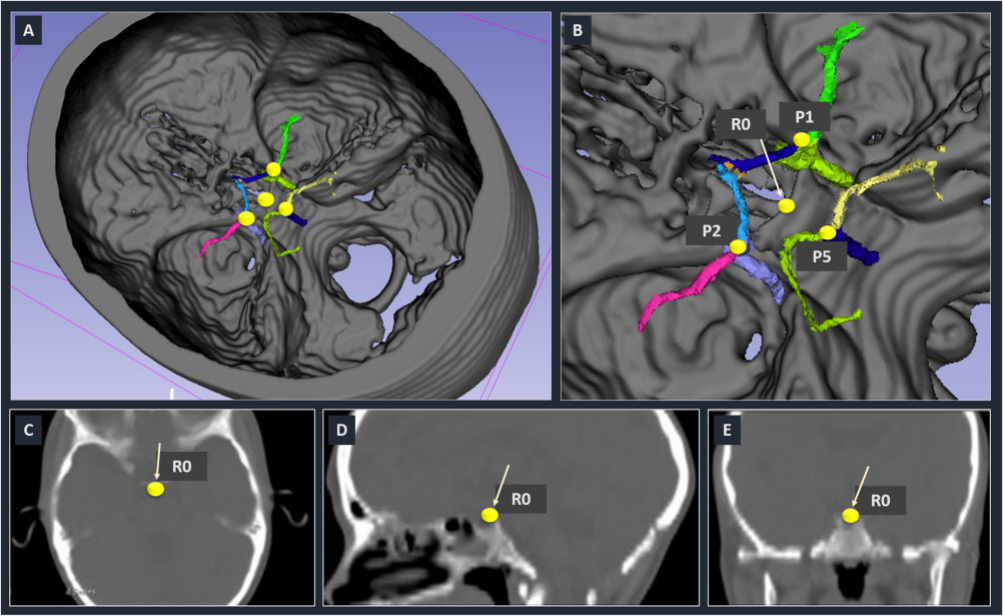
**Rigid registration of the Circle of Willis on a common skull.** (**A**) Global three-dimensional representation. (**B**) Zoomed-in view on the Circle of Willis region. Three (P1, P2 and P5) of the five landmarks defined for distance calculations can be distinguished. The reference landmark (R0) is also shown. (**C**–**E**) Illustrate the anatomical location of R0 in the skull. The white arrow is pointing to R0.

Thus, we obtained The X, Y, Z coordinates of each landmark. Similarly, we defined R0, as being the apex of the posterior lip of the turcica sella as a common reference landmark (Fig. 2). The distances between R0 and the landmarks L1 to L5, as well as the distances between landmarks, and the perimeter L1-L2-L5, considered as a surrogate of the Circle of Willis circumference (Fig. 2 and Supplementary Fig. 2) were calculated for each of the 38 subjects.

### Statistical methods

Quantile to quantile plots (Q-Q plots) and Shapiro-Wilk test were used to investigate the normality of the distribution of the different calculated distances.^15^

In addition to the mean and standard deviation, the coefficient of variation of each distance was calculated to describe the relative dispersion of these measurements.^16^

Generalized linear models were used to investigate the variability and the predictors of distances between the different landmarks, age and gender were investigated as covariates.^17^

We studied the variability of distances between the skull base and the five landmarks as first step, then the variability of distances within the Circle of Willis, we calculated the ratio L1-L3/L2-L4 as an indicator of the symmetry (as they measure the same distance in the right and left ICA).

### Data availability statement

Age, weight, X, Y and Z coordinates of the five landmarks for the 38 subject data are available in the Supplementary material.

Further data used in this work will be available upon reasonable request to the corresponding author.

## Results

Distances from R0 to any of the L1-L5 landmarks ranged from 7.0 to 24.4 mm (Table 1). No significant deviance from normality was observed in the distribution of these five distances, as shown by Q-Q plots (Supplementary Fig. 3) and the Shapiro-Wilk test. When considering both gender together, the variability was remarkably low, the coefficients of variation ranging from 0.15 to 0.22, the findings remaining similar when considering each gender separately. Overall, L1, L2, L3 and L4 were almost equidistant from R0, L5 being lightly nearest for R0 (15% lesser than the average distance) than the mean of the four other landmarks (16.1 mm).

**Table 1:** Distribution parameters of distances between landmarks in 38 subjects

	Mean	Median	Standard deviation	Coefficient of variation	Range	Shapiro test for normality
Whole sample (*n* = 38)						
R0-L1	15.5	15.4	2.7	0.17	10.7–21.9	0.60
R0-L2	16.7	16.0	2.9	0.17	11.1–22.4	0.27
R0-L3	15.3	14.8	2.8	0.18	10.6–20.9	0.33
R0-L4	17.0	16.7	2.6	0.15	11.5–24.4	0.41
R0-L5	13.6	13.6	3.0	0.22	7.0–19.6	0.88
L1-L2	26.8	25.9	3.4	0.13	21.4–36.8	0.03
L1-L3	16.4	16.3	2.5	0.16	12.2–22.1	0.41
L1-L5	18.8	18.8	2.7	0.14	12.8–23.3	0.46
L2-L4	15.8	15.6	2.3	0.14	11.7–22.4	0.21
L2-L5	18.4	18.3	2.8	0.15	12.8–24.1	0.47
(L1-L3)/(L2-L4)	1.04	1.03	0.1	0.10	0.80–1.2	0.82
L1-L2-L5	64.0	63.5	7.1	0.11	46.9–83.7	0.81
Females (*n* = 19)						
R0-L1^a^	14.4	14.3	2.3	0.16	10.7–21.4	0.05
R0-L2	16.5	15.8	2.9	0.18	12.2–22.4	0.05
R0-L3	15.2	14.7	2.5	0.16	11.4–20.9	0.55
R0-L4	17.2	16.3	3.0	0.17	13.0–14.4	0.24
R0-L5	13.6	14.3	2.7	0.20	8.0–18.0	0.83
L1-L2	26.8	26.0	3.6	0.14	22.0–36.8	0.04
L1-L3	16.0	15.2	2.9	0.18	12.2–21.2	0.13
L1-L5	18.8	18.1	2.5	0.13	15.1–23.3	0.07
L2-L4	15.4	15.0	2.5	0.16	11.7–20.7	0.44
L2-L5	18.6	18.1	3.1	0.17	13.5–24.1	0.24
(L1-L3)/(L2-LP4)	1.05	1.03	0.12	0.11	0.8–1.2	0.47
L1-L2-L5	64.2	64.0	7.4	0.12	54.0–83.7	0.25
Males (*n* = 19)						
R0-L1^a^	16.5	16.7	2.6	0.16	12.0–21.9	0.81
R0-L2	17.0	17.0	2.8	0.16	11.1–22.3	1
R0-L3	15.3	14.9	3.1	0.20	10.6–20.3	0.18
R0-L4	16.8	16.7	2.2	0.13	11.5–20.3	0.42
R0-L5	13.6	13.5	3.4	0.25	7.0–19.6	0.76
L1-L2	26.8	25.7	3.3	0.12	21.4–32.5	0.29
L1-L3	16.8	17.0	2.1	0.13	13.4–22.1	0.46
L1-L5	18.7	19.1	2.9	0.15	12.8–23.2	0.81
L2-L4	16.3	16.1	2.0	0.12	13.4–22.4	0.03
L2-L5	18.2	18.5	2.7	0.15	12.8–22.3	0.66
(L1-L3)/(L2-L4)	1.04	1.04	0.1	0.09	0.9–1.2	0.88
L1-L2-L5	63.7	63.1	7.0	0.11	46.9–76.1	0.78

R0: Posterior clinoid processes of the sella turcica.

L1: Intersection of right internal carotid artery and middle and anterior cerebral artery.

L2: Intersection of left internal carotid artery and middle and anterior cerebral artery.

L3: Top of the right cavernous sinus.

L4: Top of the left cavernous sinus.

L5: End of basilar artery and birth of the right and left posterior cerebral arteries.

a *P*-value for difference between genders = 0.01.

Distances between two landmarks within the Circle of Willis have a remarkably low variation, the coefficients of variation ranging from 0.12 to 0.18. The ratio between two symmetrical distances, here L1-L3 and L2-L4 was consistently close to one, and the (L1-L2-L5) perimeter expresses a low variability, it’s coefficient of variation being the lowest. The average length of the perimeter was 64.0 mm, 64.2 for females and 63.7 for males (SD 7 and 7.4, respectively), ranging from 46.9 to 83.7 mm.

**Figure 3:**
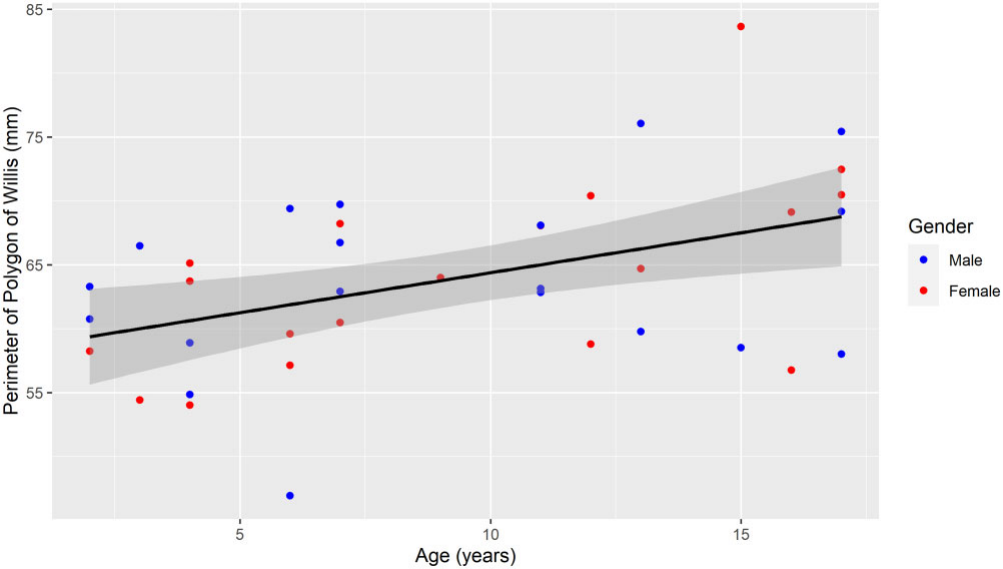
**Scatter plot of the variation of the Circle of Willis perimeter according to age in males and females, respectively.** The line represents the growth of the Circle of Willis perimeter with age, obtained by linear regression on data for the entire population. The dark grey shadow represents the 95% confidence intervals limits.

**Figure 4:**
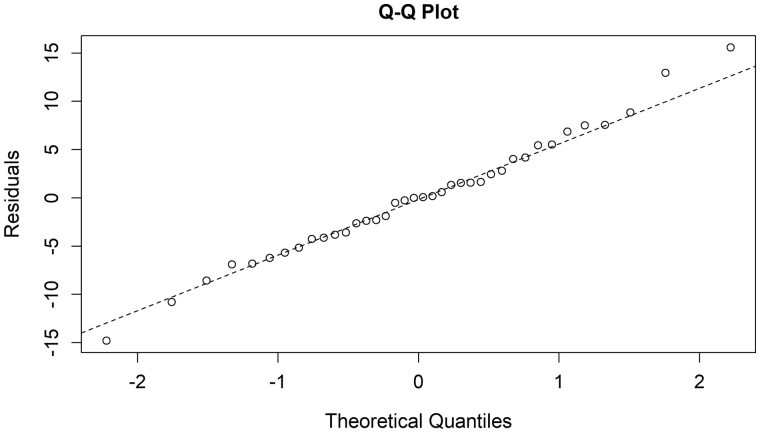
**Quantile to Quantile plot of the residuals of the linear regression model for the growth of the Circle of Willis perimeter with age**.

In a multivariate model, the Circle of Willis circumference surrogate (L1-L2-L5) was significantly linked to age, the linear regression coefficient was 0.62 (SD = 0.21, *P* = 0.005), but not influenced by gender (*P* = 0.9) (Fig. 3). The increase in the perimeter for age increasing, was most important in the antero-posterior axis (*P* = 0.02 and 0.001, respectively for L1-L5 and L2-5) than in the lateral axis (L1-L2, *P* = 0.2) (Table 2, Supplementary Fig. 3). In this model, no significant deviance from normality was observed in the distribution of residuals, as shown in (Fig. 4).

**Table 2:** Role of age and gender in the variance of distance between some key landmarks in the circle of Willis in 38 subjects

	Age			Gender			*P*-value for interaction between age and gender^c^
	Coefficient	SD	*P*-value^a^	Coefficient	SD	*P*-value^b^	
R0-L1	0.094	0.078	0.2	−2.20	0.79	0.009	0.6
R0-L2	0.073	0.094	0.4	−0.51	0.94	0.6	0.9
R0-L3	0.12	0.091	0.2	−0.18	0.92	0.8	0.06
R0-L4	0.083	0.086	0.3	0.41	0.84	0.6	0.2
R0-L5	−0.016	0.099	0.9	0.0025	1.01	0.9	0.6
L1-L3	−0.017	0.083	0.9	−0.74	0.84	0.4	0.1
L2-L4	0.0097	0.075	0.9	−0.86	0.75	0.3	0.2
(L1-L3)/(L2-L4)	−0.00052	0.0034	0.9	0.0098	0.034	0.8	0.3
L1-L2-L5	0.62	0.21	0.005	0.18	2.12	0.9	0.3
L1-L2	0.15	0 11	0.2	−0.084	1.11	0.9	0.4
L1-L5	0.27	0.076	0.001	−0.026	0.77	0.9	0.6
L2-L5	0.20	0.087	0.02	0.29	0.88	0.7	0.2

R0: Posterior clinoid processes of the sella turcica.

L1: Intersection of right internal carotid artery and middle and anterior cerebral artery.

L2: Intersection of left internal carotid artery and middle and anterior cerebral artery.

L3: Top of the right cavernous sinus.

L4: Top of the left cavernous sinus.

L5: End of basilar artery and birth of the right and left posterior cerebral arteries.

^a^ *p*-value for age difference.

^b^ *p*-value for gender difference.

^c^ *p*-value for interaction.

## Discussion

Our study showed a low variability for the constituent arteries of Circle of Willis, as the distance between each of the five landmarks chosen to represent the shape of this structure. The Circle of Willis grows in an isotropic way, the variation coefficients being very low range 0.10–0.17. When considering the distance with the bone landmark the variation coefficients were slightly higher 0.15–0.22, but remained globally low. As a general matter, these distances followed a normal distribution.

In a linear regression, a significant increase in the Circle of Willis perimeter of 0.62 mm in average for each year was observed, which has to be correlated to the natural growth of the skull bones, since the development of the cerebral arteries, especially the ICA, seems closely related to that of the skull bones. We did not observe significant effect of gender. Nevertheless, the small number of patients did not permit us to deeply investigate the shape of the relation between gender, age and Circle of Willis parameters.

The data published by the World Health Organization on cranial perimeter shows that most of the growth takes place between birth and 2 years, the average cranial perimeter increasing from 339–345 to 472–482 mm. After 2 years, the growth rate is significantly lower and reaches 499–507 mm at 5 years,^18^ period during which the average annual increase of the median cranial perimeter is of 1.6% of its value.^18^ In another study, performed by the NIH, a linear growth of the total brain volume for males and a quadratic growth for females in a population of 4–18 years, the total brain volume globally increasing by 2.03% per year, i.e. an increase of 1.27% brain perimeter.^19^ This last value is similar to the annual increase of about 1% (0.6 mm/60 mm) we observed in our sample in which age ranged from 2 to 18.

In this work, the registration of the Circle of Willis in the coordinate system centred on the skull bone foramina, makes our results independent of the position of the child head and accounts for the possible movement of rotation, flexion and extension that are frequent during the MRI procedure, which can vary according to the position during radiotherapy treatment. On the other hand, the registration as well as identification of the 5 landmarks on each one of the 38 MRI are done manually and are therefore subject to the subjectivity of the operator, an inconvenient that could be bypassed with the development of automatic and semi-automatic registration and contouring methods.^20^ Additionally, our work was limited to five anatomically and easily recognizable key landmarks, and could be improved by including most of the Circle of Willis shape and pixels distributed over the entire volume of Circle of Willis. In this work, we were only interested in the topographic variability of the Circle of Willis constituent arteries. Hence we included only MRIs with no anomalies or anatomical variant, as we are not interested on the morphological variability of the Willis Polygon largely described in the literature. Lastly, most subjects in our sample had an MRI motivated by the suspicion of a stroke in a context of sickle cell disease. Up to now, no study has shown specific anatomical characteristic of the Circle of Willis in these patients.^21^

In our knowledge, this is the only study analysing the topographic variability of the Willis Polygon. It was motivated by reports of long-term cerebrovascular toxicity in the adulthood of paediatric cancer survivors, based on retrospective dosimetry, which showed a correlation between doses delivered to the Circle of Willis and long-term cerebrovascular toxicities. This study shows that in anthropomorphic phantoms used for retrospective dose-reconstruction, the Circle of Willis can be represented by a mean shape derived from segmented MRIs, this process being feasible and reliable.

## Supplementary material

Supplementary material is available at *Brain Communications* online.

## Funding

W.Z. received support as recipient of the DUERTECC/EURONCO education grant (Diplome Universitaire Européen de Recherche Translationelle et Clinique en Cancerologie – no grant number applicable). This project was supported by the Foundation ARC for Cancer Resarch (grant no. Pop-HaRC 201401208) and the “Mr Robot” PAIR Research Program (grant no. INCa-Fondation ARC-LNCC 11909).

The funding source had no role in the preparation of this article.

## Competing interests

The authors report no competing interests.

## Supplementary Material

fcab055_Supplementary_Data

## Abbreviations

ICA =: the internal carotid arteries
