# Supplementary material for: Topographic variability of the normal circle of Willis anatomy on a paediatric population
Source: Brain Commun. 2021 Apr 3;3(2):fcab055. doi: 10.1093/braincomms/fcab055 (PMC8204365; doi:10.1093/braincomms/fcab055)

**Supplementary material**

**Supplementary Table 1:**

Topographic coordinates of posterior clinoid processes of sella turcica and of 5 topographic landmarks of the Circle of Willis.

| Patient | Age (year) | Weight | Gender | Points | X | Y | Z |
| --- | --- | --- | --- | --- | --- | --- | --- |
| 0 |  |  |  | R0 | 1,4731 | -10,6774 | -106,193 |
|  |  |  |  |  |  |  |  |
| 1 | 3 | 11 | female | L1 | 12,4611 | -8,67756 | -102,596 |
| 1 | 3 | 11 | female | L2 | -9,4761 | -8,07325 | -101,372 |
| 1 | 3 | 11 | female | L3 | 12,0857 | -1,74287 | -112,591 |
| 1 | 3 | 11 | female | L4 | -8,38509 | -0,19162 | -110,187 |
| 1 | 3 | 11 | female | L5 | 0,071609 | -20,1301 | -103,502 |
|  |  |  |  |  |  |  |  |
| 2 | 4 | 15 | female | L1 | 10,4379 | -10,4373 | -95,5877 |
| 2 | 4 | 15 | female | L2 | -13,6344 | -11,6794 | -99,4575 |
| 2 | 4 | 15 | female | L3 | 9,73716 | -1,41712 | -110,866 |
| 2 | 4 | 15 | female | L4 | -12,535 | -3,25719 | -111,838 |
| 2 | 4 | 15 | female | L5 | -3,05978 | -19,1669 | -95,6015 |
|  |  |  |  |  |  |  |  |
| 3 | 2 | 13 | female | L1 | 11,3272 | -14,5215 | -104,535 |
| 3 | 2 | 13 | female | L2 | -13,0524 | -12,9634 | -105,975 |
| 3 | 2 | 13 | female | L3 | 9,65831 | -5,36062 | -116,618 |
| 3 | 2 | 13 | female | L4 | -12,3915 | -2,67734 | -117,003 |
| 3 | 2 | 13 | female | L5 | -0,3788 | -25,2685 | -106,981 |
|  |  |  |  |  |  |  |  |
| 4 | 4 | 21 | female | L1 | 16,5266 | -15,89 | -102,775 |
| 4 | 4 | 21 | female | L2 | -12,3088 | -14,4512 | -99,5532 |
| 4 | 4 | 21 | female | L3 | 12,2377 | -6,49468 | -111,409 |
| 4 | 4 | 21 | female | L4 | -10,7395 | -3,63181 | -111,632 |
| 4 | 4 | 21 | female | L5 | -0,21895 | -25,5306 | -102,986 |
|  |  |  |  |  |  |  |  |
| 5 | 4 | 22 | female | L1 | 10,4815 | -9,46248 | -94,8458 |
| 5 | 4 | 22 | female | L2 | -17,9173 | -8,87284 | -98,5418 |
| 5 | 4 | 22 | female | L3 | 11,1829 | 1,55192 | -112,335 |
| 5 | 4 | 22 | female | L4 | -13,5806 | 2,41263 | -114,742 |
| 5 | 4 | 22 | female | L5 | -5,91314 | -18,7189 | -93,8474 |
|  |  |  |  |  |  |  |  |
| 6 | 2 | 12 | male | L1 | 16,5663 | -0,93451 | -101,864 |
| 6 | 2 | 12 | male | L2 | -8,67082 | -1,86393 | -101,744 |
| 6 | 2 | 12 | male | L3 | 11,8953 | 2,44146 | -115,07 |
| 6 | 2 | 12 | male | L4 | -5,99259 | 3,70634 | -115,51 |
| 6 | 2 | 12 | male | L5 | 1,39393 | -13,645 | -99,9005 |
|  |  |  |  |  |  |  |  |
| 7 | 2 | 17 | male | L1 | 16,1692 | -14,9126 | -96,4265 |
| 7 | 2 | 17 | male | L2 | -13,3973 | -14,0654 | -98,0901 |
| 7 | 2 | 17 | male | L3 | 8,73166 | -3,83917 | -110,947 |
| 7 | 2 | 17 | male | L4 | -9,79556 | -2,92088 | -111,056 |
| 7 | 2 | 17 | male | L5 | 0,812344 | -22,2171 | -99,1878 |
|  |  |  |  |  |  |  |  |
| 8 | 4 | 20 | male | L1 | 11,2971 | -9,49798 | -97,5886 |
| 8 | 4 | 20 | male | L2 | -13,4163 | -9,0802 | -98,1076 |
| 8 | 4 | 20 | male | L3 | 7,55061 | -0,37726 | -108,736 |
| 8 | 4 | 20 | male | L4 | -9,50457 | 1,19403 | -109,435 |
| 8 | 4 | 20 | male | L5 | -0,55296 | -20,7348 | -100,684 |
|  |  |  |  |  |  |  |  |
| 9 | 4 | 13 | male | L1 | 14,9532 | -8,5622 | -98,1334 |
| 9 | 4 | 13 | male | L2 | -8,41787 | -7,78057 | -97,7033 |
| 9 | 4 | 13 | male | L3 | 10,4185 | 0,903376 | -112,951 |
| 9 | 4 | 13 | male | L4 | -9,0324 | 1,3582 | -111,62 |
| 9 | 4 | 13 | male | L5 | 3,23425 | -18,5391 | -96,1698 |
|  |  |  |  |  |  |  |  |
| 10 | 3 | 12 | male | L1 | 14,8803 | -14,1214 | -99,2049 |
| 10 | 3 | 12 | male | L2 | -11,8881 | -13,7119 | -98,9355 |
| 10 | 3 | 12 | male | L3 | 15,786 | -3,62655 | -113,453 |
| 10 | 3 | 12 | male | L4 | -10,4861 | -2,38352 | -111,786 |
| 10 | 3 | 12 | male | L5 | -0,74834 | -27,9822 | -102,813 |
|  |  |  |  |  |  |  |  |
| 11 | 9 | 30 | female | L1 | 12,8922 | -13,7242 | -98,1149 |
| 11 | 9 | 30 | female | L2 | -11,5548 | -12,1844 | -100,112 |
| 11 | 9 | 30 | female | L3 | 13,1552 | -2,84764 | -110,101 |
| 11 | 9 | 30 | female | L4 | -8,143 | -2,96099 | -110,466 |
| 11 | 9 | 30 | female | L5 | 3,48646 | -28,0298 | -101,7 |
|  |  |  |  |  |  |  |  |
| 12 | 6 | 20 | female | L1 | 13,8902 | -12,8769 | -97,7206 |
| 12 | 6 | 20 | female | L2 | -12,4082 | -14,0894 | -99,2715 |
| 12 | 6 | 20 | female | L3 | 10,4658 | -3,58898 | -110,095 |
| 12 | 6 | 20 | female | L4 | -11,3923 | -3,34161 | -109,543 |
| 12 | 6 | 20 | female | L5 | 0,145353 | -23,578 | -99,0609 |
|  |  |  |  |  |  |  |  |
| 13 | 7 | 40 | female | L1 | 13,8458 | -12,6645 | -96,4142 |
| 13 | 7 | 40 | female | L2 | -17,2258 | -14,6689 | -99,2421 |
| 13 | 7 | 40 | female | L3 | 14,5307 | 2,82049 | -110,906 |
| 13 | 7 | 40 | female | L4 | -12,2033 | -0,358 | -108,603 |
| 13 | 7 | 40 | female | L5 | 3,25596 | -22,1541 | -101,334 |
|  |  |  |  |  |  |  |  |
| 14 | 7 | 20 | female | L1 | 10,7562 | -8,523 | -99,9643 |
| 14 | 7 | 20 | female | L2 | -13,5188 | -8,71129 | -101,461 |
| 14 | 7 | 20 | female | L3 | 7,78507 | -1,18571 | -109,623 |
| 14 | 7 | 20 | female | L4 | -10,2062 | -0,07482 | -109,817 |
| 14 | 7 | 20 | female | L5 | -0,9047 | -20,1495 | -107,466 |
|  |  |  |  |  |  |  |  |
| 15 | 6 | 17 | female | L1 | 12,3069 | -7,01952 | -100,455 |
| 15 | 6 | 17 | female | L2 | -11,5295 | -7,03353 | -100,139 |
| 15 | 6 | 17 | female | L3 | 6,95148 | -1,70688 | -110,689 |
| 15 | 6 | 17 | female | L4 | -7,24142 | -0,46602 | -108,84 |
| 15 | 6 | 17 | female | L5 | -1,11154 | -17,9687 | -104,055 |
|  |  |  |  |  |  |  |  |
| 16 | 6 | 25 | male | L1 | 8,45082 | -25,4899 | -109,582 |
| 16 | 6 | 25 | male | L2 | -12,7845 | -23,3449 | -109,901 |
| 16 | 6 | 25 | male | L3 | 8,79037 | -10,5268 | -114,299 |
| 16 | 6 | 25 | male | L4 | -11,1615 | -8,30757 | -115,533 |
| 16 | 6 | 25 | male | L5 | -2,34377 | -26,9905 | -116,286 |
|  |  |  |  |  |  |  |  |
| 17 | 7 | 30 | male | L1 | 16,32 | -14,7663 | -90,6178 |
| 17 | 7 | 30 | male | L2 | -15,9952 | -12,4841 | -92,474 |
| 17 | 7 | 30 | male | L3 | 13,3351 | 0,560729 | -106,298 |
| 17 | 7 | 30 | male | L4 | -9,81358 | 1,28643 | -109,039 |
| 17 | 7 | 30 | male | L5 | 3,50149 | -18,9074 | -93,2792 |
|  |  |  |  |  |  |  |  |
| 18 | 7 | 30 | male | L1 | 18,5597 | -10,1554 | -102,002 |
| 18 | 7 | 30 | male | L2 | -10,2638 | -5,66167 | -101,569 |
| 18 | 7 | 30 | male | L3 | 15,1085 | -1,42662 | -115,748 |
| 18 | 7 | 30 | male | L4 | -9,04839 | -0,23867 | -113,933 |
| 18 | 7 | 30 | male | L5 | 1,24095 | -21,6891 | -102,158 |
|  |  |  |  |  |  |  |  |
| 19 | 6 | 25 | male | L1 | 13,6607 | -14,6402 | -93,6982 |
| 19 | 6 | 25 | male | L2 | -14,4562 | -9,01759 | -94,2528 |
| 19 | 6 | 25 | male | L3 | 9,99085 | -3,9329 | -106,4 |
| 19 | 6 | 25 | male | L4 | -11,0892 | -1,22843 | -105,526 |
| 19 | 6 | 25 | male | L5 | -2,04525 | -25,1719 | -99,3005 |
|  |  |  |  |  |  |  |  |
| 20 | 7 | 27 | male | L1 | 11,6155 | -12,868 | -100,205 |
| 20 | 7 | 27 | male | L2 | -11,9821 | -12,8107 | -99,0316 |
| 20 | 7 | 27 | male | L3 | 13,6194 | -0,52261 | -113,089 |
| 20 | 7 | 27 | male | L4 | -10,5581 | 0,347169 | -111,234 |
| 20 | 7 | 27 | male | L5 | 0,621596 | -28,4947 | -100,448 |
|  |  |  |  |  |  |  |  |
| 21 | 11 | 37 | female | L1 | 12,2218 | -6,33031 | -99,6469 |
| 21 | 11 | 37 | female | L2 | -11,3287 | -6,89661 | -99,182 |
| 21 | 11 | 37 | female | L3 | 10,2265 | 0,176999 | -113,016 |
| 21 | 11 | 37 | female | L4 | -11,7244 | 0,429114 | -111,737 |
| 21 | 11 | 37 | female | L5 | -1,45091 | -20,8383 | -111,72 |
|  |  |  |  |  |  |  |  |
| 22 | 12 | 42 | female | L1 | 16,0012 | -10,0331 | -101,488 |
| 22 | 12 | 42 | female | L2 | -8,43346 | -10,6759 | -97,6692 |
| 22 | 12 | 42 | female | L3 | 15,0907 | -2,80098 | -112,697 |
| 22 | 12 | 42 | female | L4 | -11,872 | -3,7765 | -112,556 |
| 22 | 12 | 42 | female | L5 | 2,37931 | -22,7905 | -114,135 |
|  |  |  |  |  |  |  |  |
| 23 | 12 | 33 | female | L1 | 14,1756 | -17,1742 | -104,944 |
| 23 | 12 | 33 | female | L2 | -11,8224 | -16,3899 | -106,798 |
| 23 | 12 | 33 | female | L3 | 13,5466 | -6,03942 | -113,161 |
| 23 | 12 | 33 | female | L4 | -11,6603 | -4,58137 | -113,617 |
| 23 | 12 | 33 | female | L5 | 0,418225 | -25,3136 | -110,822 |
|  |  |  |  |  |  |  |  |
| 24 | 13 | 45 | female | L1 | 15,6681 | -11,0593 | -101,192 |
| 24 | 13 | 45 | female | L2 | -10,2376 | -12,938 | -99,3815 |
| 24 | 13 | 45 | female | L3 | 11,2889 | -0,90619 | -110,998 |
| 24 | 13 | 45 | female | L4 | -7,9117 | -1,75101 | -110,092 |
| 24 | 13 | 45 | female | L5 | 2,05699 | -22,5619 | -109,883 |
|  |  |  |  |  |  |  |  |
| 25 | 11 | 45 | male | L1 | 12,2792 | -11,871 | -100,127 |
| 25 | 11 | 45 | male | L2 | -13,4468 | -11,259 | -100,24 |
| 25 | 11 | 45 | male | L3 | 14,8664 | -0,22876 | -113,883 |
| 25 | 11 | 45 | male | L4 | -14,1151 | -0,10041 | -113,561 |
| 25 | 11 | 45 | male | L5 | -0,10856 | -24,2217 | -105,069 |
|  |  |  |  |  |  |  |  |
| 26 | 11 | 42 | male | L1 | 12,2272 | -0,01931 | -94,3461 |
| 26 | 11 | 42 | male | L2 | -12,8347 | -2,10842 | -95,2629 |
| 26 | 11 | 42 | male | L3 | 9,88648 | 5,42605 | -109,619 |
| 26 | 11 | 42 | male | L4 | -10,6814 | 5,37959 | -108,491 |
| 26 | 11 | 42 | male | L5 | -1,01651 | -14,9874 | -92,9493 |
|  |  |  |  |  |  |  |  |
| 27 | 13 | 89 | male | L1 | 15,9122 | -6,34557 | -102,277 |
| 27 | 13 | 89 | male | L2 | -15,7173 | -8,336 | -102,075 |
| 27 | 13 | 89 | male | L3 | 16,7984 | -1,21463 | -115,517 |
| 27 | 13 | 89 | male | L4 | -14,2689 | -1,48453 | -115,204 |
| 27 | 13 | 89 | male | L5 | 1,03302 | -20,9149 | -109,653 |
|  |  |  |  |  |  |  |  |
| 28 | 13 | 30 | male | L1 | 8,83686 | -7,15242 | -95,6225 |
| 28 | 13 | 30 | male | L2 | -15,365 | -7,6861 | -99,1914 |
| 28 | 13 | 30 | male | L3 | 9,75869 | 0,456016 | -111,182 |
| 28 | 13 | 30 | male | L4 | -11,9002 | 0,787975 | -111,702 |
| 28 | 13 | 30 | male | L5 | -1,74524 | -17,914 | -104,424 |
|  |  |  |  |  |  |  |  |
| 29 | 11 | 50 | male | L1 | 14,3662 | -17,1569 | -99,1108 |
| 29 | 11 | 50 | male | L2 | -16,7726 | -14,7137 | -96,9141 |
| 29 | 11 | 50 | male | L3 | 13,113 | -3,85444 | -110,078 |
| 29 | 11 | 50 | male | L4 | -13,7299 | -2,92599 | -108,295 |
| 29 | 11 | 50 | male | L5 | 0,33128 | -24,4075 | -102,846 |
|  |  |  |  |  |  |  |  |
| 30 | 16 |  | female | L1 | 9,70528 | -17,2785 | -99,3566 |
| 30 | 16 |  | female | L2 | -13,4891 | -16,6364 | -100,735 |
| 30 | 16 |  | female | L3 | 13,039 | -3,58782 | -112,684 |
| 30 | 16 |  | female | L4 | -15,089 | -1,92711 | -115,265 |
| 30 | 16 |  | female | L5 | -2,88129 | -27,4738 | -105,933 |
|  |  |  |  |  |  |  |  |
| 31 | 17 |  | female | L1 | 13,7721 | -14,0602 | -96,4418 |
| 31 | 17 |  | female | L2 | -15,8055 | -12,9235 | -96,8083 |
| 31 | 17 |  | female | L3 | 13,4924 | -1,42203 | -113,23 |
| 31 | 17 |  | female | L4 | -11,3351 | -1,69169 | -110,191 |
| 31 | 17 |  | female | L5 | 0,290393 | -26,394 | -102,293 |
|  |  |  |  |  |  |  |  |
| 32 | 15 |  | female | L1 | 19,2308 | -10,8792 | -94,2257 |
| 32 | 15 |  | female | L2 | -17,4618 | -7,80777 | -94,6296 |
| 32 | 15 |  | female | L3 | 11,6256 | -0,8792 | -104,972 |
| 32 | 15 |  | female | L4 | -12,1449 | 0,21033 | -104,116 |
| 32 | 15 |  | female | L5 | 0,770037 | -21,5979 | -102,156 |
|  |  |  |  |  |  |  |  |
| 33 | 16 |  | female | L1 | 12,1806 | -7,96198 | -98,5876 |
| 33 | 16 |  | female | L2 | -18,3021 | -6,65085 | -100,672 |
| 33 | 16 |  | female | L3 | 15,8164 | 3,10843 | -112,62 |
| 33 | 16 |  | female | L4 | -16,3198 | 4,89413 | -112,015 |
| 33 | 16 |  | female | L5 | -3,92461 | -18,9162 | -98,2435 |
|  |  |  |  |  |  |  |  |
| 34 | 17 |  | female | L1 | 15,1491 | -7,12433 | -101,189 |
| 34 | 17 |  | female | L2 | -13,4767 | -7,99102 | -104,112 |
| 34 | 17 |  | female | L3 | 14,043 | -0,00352 | -114,075 |
| 34 | 17 |  | female | L4 | -14,3719 | 0,131365 | -114,971 |
| 34 | 17 |  | female | L5 | -0,20541 | -23,1482 | -107,875 |
|  |  |  |  |  |  |  |  |
| 35 | 17 |  | male | L1 | 15,4687 | -8,63135 | -96,7132 |
| 35 | 17 |  | male | L2 | -7,4652 | -11,8582 | -99,6487 |
| 35 | 17 |  | male | L3 | 11,7505 | -2,03349 | -112,642 |
| 35 | 17 |  | male | L4 | -9,28248 | -1,29147 | -112,365 |
| 35 | 17 |  | male | L5 | 0,978605 | -21,8826 | -103,853 |
|  |  |  |  |  |  |  |  |
| 36 | 17 |  | male | L1 | 19,3232 | -10,9375 | -97,2527 |
| 36 | 17 |  | male | L2 | -11,2255 | -9,21615 | -96,912 |
| 36 | 17 |  | male | L3 | 12,2929 | -0,91649 | -104,828 |
| 36 | 17 |  | male | L4 | -7,36763 | 0,10535 | -105,681 |
| 36 | 17 |  | male | L5 | 2,0502 | -21,945 | -108,238 |
|  |  |  |  |  |  |  |  |
| 37 | 15 |  | male | L1 | 12,2025 | -16,8313 | -95,0128 |
| 37 | 15 |  | male | L2 | -11,9207 | -18,2667 | -97,3011 |
| 37 | 15 |  | male | L3 | 11,2735 | -6,74366 | -106,932 |
| 37 | 15 |  | male | L4 | -8,37241 | -4,89422 | -107,829 |
| 37 | 15 |  | male | L5 | -0,96798 | -27,8456 | -102,579 |
|  |  |  |  |  |  |  |  |
| 38 | 17 |  | male | L1 | 15,2463 | -10,9042 | -96,5168 |
| 38 | 17 |  | male | L2 | -11,8437 | -12,1689 | -96,3893 |
| 38 | 17 |  | male | L3 | 12,6668 | -1,46475 | -105,61 |
| 38 | 17 |  | male | L4 | -12,4008 | -0,3176 | -105,928 |
| 38 | 17 |  | male | L5 | -0,34799 | -23,2794 | -107,338 |

**Supplementary Figure 1**

3D representation of the result after automated growing region segmentation


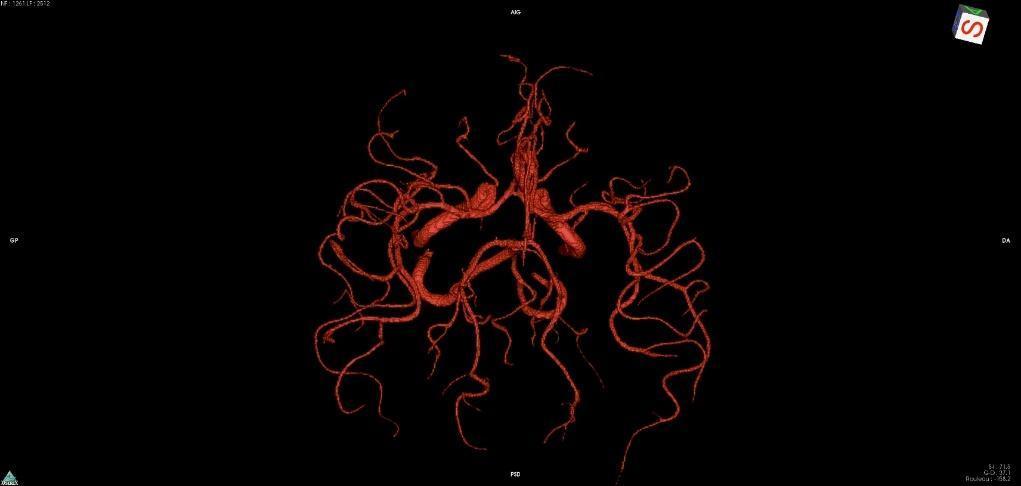


**Supplementary Figure 2:**

3D representation of the segmented Willis circle


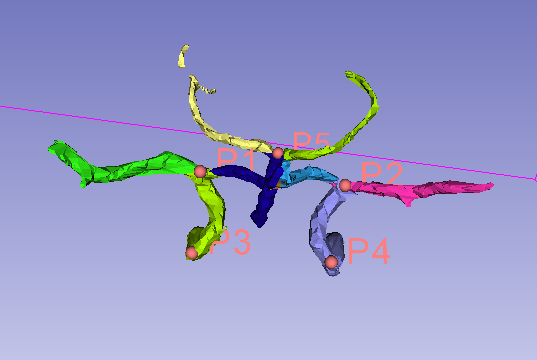


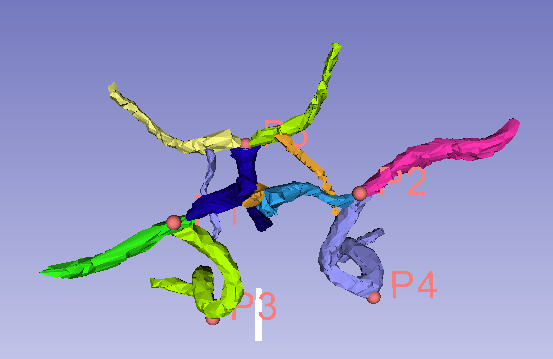


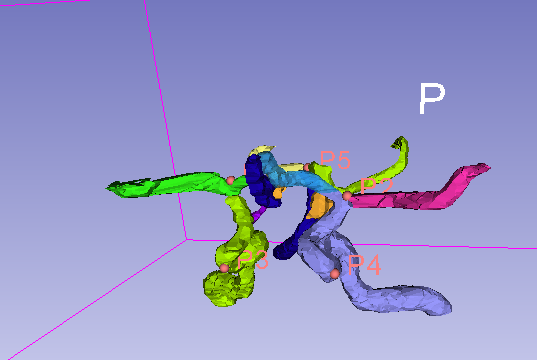


**Supplementary Figure 3:**

Q-Q plots of the distances between the reference landmark and the five circle of Willis landmarks


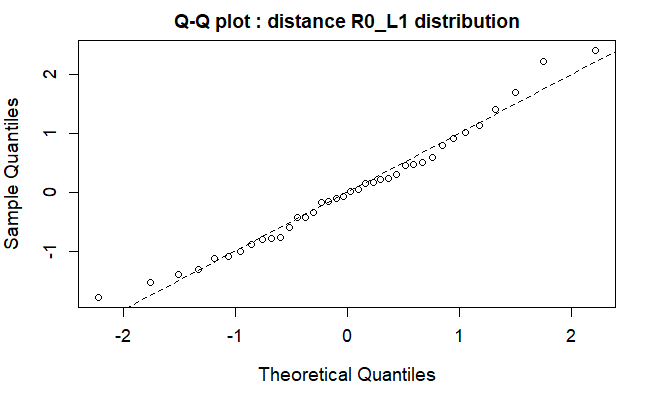

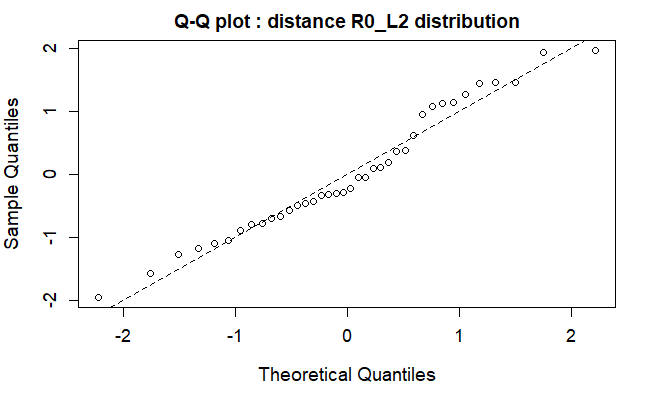

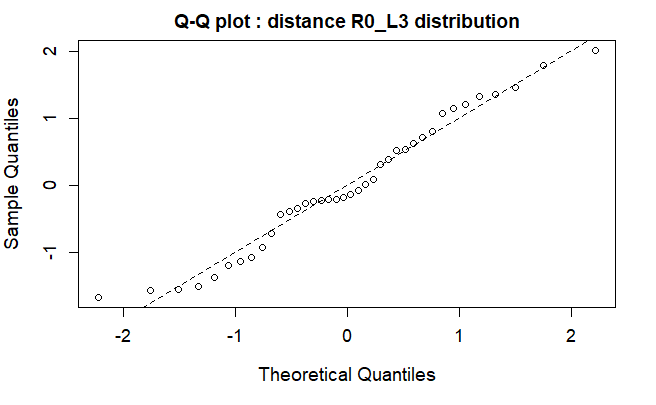

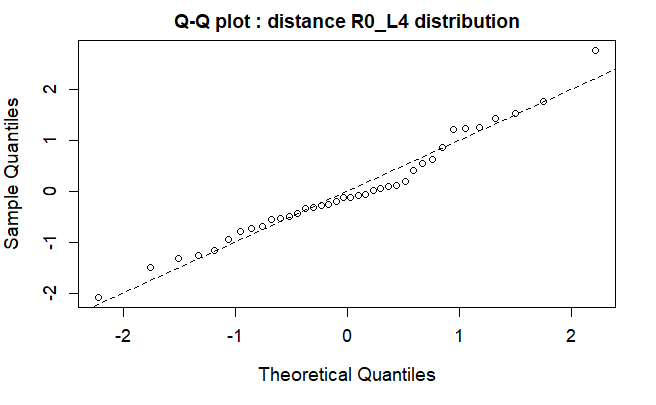

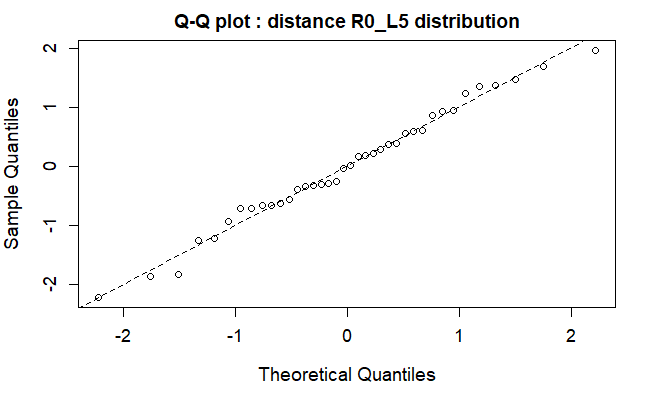

Supplement: fcab055_Supplementary_Data [file fcab055_supplementary_data.zip › Supplementary_material_final.docx]
