# Supplementary material for: Topographic variability of the normal circle of Willis anatomy on a paediatric population
Source: Brain Commun. 2021 Apr 3;3(2):fcab055. doi: 10.1093/braincomms/fcab055 (PMC8204365; doi:10.1093/braincomms/fcab055)
Supplement: fcab055_Supplementary_Data [file fcab055_supplementary_data.zip › Review_files.zip!/Original Submission-Authors Response.pdf]

Reviewer: 1

Thank you for an interesting paper. I think that the methods are sound and the results are useful. I do think that the language in the paper (with Willis' circle rather than circle of Willis) could be improved and would result in an easier read. I presume that this is because English is not the primary language of the author.

- Thank you for your interesting remarks, and your appreciation for our work.
  Our choice was to use the term "**Circle of Willis**", a referenced MeSH Term (https://www.ncbi.nlm.nih.gov/mesh/68002941) in the "Pubmed MeSH Database".

There are some copy editing changes around bracket placement that the journal staff will be able to help with.

- We have reviewed and corrected some formatting and typos.

Reviewer: 2

In this paper, the authors report inter-individual topographic variability in Circle of Willis structures in a paediatric population. I think this data is a useful addition to the literature and is of potential benefit for studies in children with brain tumors receiving radiotherapy.
General comment- the authors tend to use the term 'Willis circle' throughout the text, while this is an accepted term, to me 'Circle of Willis' sounds easier to read (this could be personal preference. There are also a few typos and full stops missing.

- Thank you for your constructive remarks, and your appreciation for our work.

- We have reviewed the redaction to adopt the "**Circle of Willis"** term throughout the text as suggested and corrected the mentioned typos.

While the relevance and importance of the study is important, I think there is quite a bit of unnecessary detail in introduction, which could be shortened.
I think the wording of the aim 'aim was to investigate the retrospective dose estimation reliability' is slightly misleading/confusing. I think the authors should provide the rationale for the study in the previous paragraph and keep the aim simple and state the aim to be 'to investigate the topographic variability.....'

- The introduction was reviewed and shortened, and we kept our aim simple as suggested.

Page 4- MRI acquisition- 2 machines with different magnet strength were used. Did the authors use the exact same scanning parameters? would the difference in scanners affect results at all

- Yes, 2 machines were used for the acquisition, they were samely used for the exploration of Willis proximal arteries in our radiology department in daily practice. The 3T one, has better performance for the study of distality.

- This would not affect our results (limited to the investigation of the proximal arteries).

Stats- no details provided re: variables used in the regression model.

- Age and gender were implemented as covariates in the regression model (we added this detail in the stat methodology).

Page 7 line 13/14, 'The average length of the perimeter was 64.0 mm (SD 7 and 7.4), ranging from 46.9 to 83.7 mm'- why two SDs are presented? what are each for?

- For the Circle of Willis perimeter length, we reported the average and the SD for both female and male, although we did not find a significant difference between females and males we preferred reporting separately both SD, as we analysed a quite small sample.
- The whole sample SD was reported in Table 1.

All figures need to have a legend describing what the images are, what they show etc. Currently no description provided at all. Also, labels used in images are not easily visible, suggest the authors use contrasting color for labels to improve visibility.

- Details were provided to the figures as suggested.
