# Supplementary material for: Topographic variability of the normal circle of Willis anatomy on a paediatric population
Source: Brain Commun. 2021 Apr 3;3(2):fcab055. doi: 10.1093/braincomms/fcab055 (PMC8204365; doi:10.1093/braincomms/fcab055)
Supplement: fcab055_Supplementary_Data [file fcab055_supplementary_data.zip › Review_files.zip!/Original Submission-Decision Letter.pdf]

Original Submission-Decision Letter

01-Dec-2020

Dear Dr. DIALLO,

Thank you for your recent submission BRAINCOM-2020-338
entitled "Topographic variability of the normal Willis circle anatomy on a pediatric population" to Brain Communications.

Your manuscript has been reviewed, and while the reviewers had largely positive comments, they have some constructive critiques that I think you could address in a minor revision of the manuscript. Therefore, I invite you to respond to the reviewer(s)' comments and revise your manuscript. The reviewer(s)' comments are included at the bottom of this message.

In addition to the reviewers' comments, please note our editorial comments added below to comply with our requirements for presentation of scientific data at Brain Communications.

Please format your revised manuscript according to our Instructions to Authors https://academic.oup.com/braincomms/pages/General_Instructions – a summary of key points is attached to help with this.

To revise your manuscript, click the link below or log into https://mc.manuscriptcentral.com/braincom and enter your Author Centre, where you will find your manuscript title listed under "Manuscripts with Decisions." Under "Actions," click on "Create a Revision." Your manuscript number has been appended to denote a revision.

*** PLEASE NOTE: This is a two-step process. After clicking on the link, you will be directed to a webpage to confirm. ***

https://mc.manuscriptcentral.com/braincom?URL_MASK=2dca4f0b5e0f4a21b01317827a41c19a

Please upload a revised manuscript with the changes you have made shown either with "track changes" or in coloured text.

When submitting your revised manuscript, you will be asked to respond to the reviewer(s) comments. Please be as specific as possible in your response to all of the comments.

The editorial team at Brain Communications also aims to increase transparency in scientific publishing and to demonstrate the value that peer review brings to the scientific process. Please therefore consider whether you are willing to have reviewer comments on your paper and your response published as supplementary data with the paper if it is accepted. This is entirely optional and will only be done if both the authors and reviewers agree. When submitting your revision you will have the opportunity to update the answer you entered previously.

IMPORTANT: Your original files are available to you when you upload your revised manuscript. Please delete any redundant files before completing the submission.

Because we are trying to facilitate timely publication of manuscripts submitted to Brain Communications, we would appreciate your revised manuscript as soon as possible. If it is not possible for you to resubmit within 3 months, the option to submit a revision will expire, and we would then consider your revised paper as a new submission. However, if you need a little more time, please get in touch and we can extend this deadline for you.

Once again, thank you for submitting your manuscript to Brain Communications and I look forward to receiving your revision.

Yours sincerely,
Prof. Richard Chin
Editor, Brain Communications

Reviewer(s)' Comments to Author:

Reviewer: 1 (Jeremy Jones)

Comments to the Author
Thank you for an interesting paper. I think that the methods are sound and the results are useful. I do think that the language in the paper (with Willis' circle rather than circle of Willis) could be improved and would result in an easier read. I presume that this is because English is not the primary language of the author. There are some copy editing changes around bracket placement that the journal staff will be able to help with.

Reviewer: 2

Comments to the Author
In this paper, the authors report inter-individual topographic variability in Circle of Willis structures in a paediatric population. I think this data is a useful addition to the literature and is of potential benefit for studies in children with brain tumors receiving radiotherapy.

General comment- the authors tend to use the term 'Willis circle' throughout the text, while this is an accepted term, to me 'Circle of Willis' sounds easier to read (this could be personal preference. There are also a few typos and full stops missing.

Introduction:
While the relevance and importance of the study is important, I think there is quite a bit of unnecessary detail in introduction, which could be shortened.

Aim:
I think the wording of the aim 'aim was to investigate the retrospective dose estimation reliability' is slightly misleading/confusing. I think the authors should provide the rationale for the study in the previous paragraph and keep the aim simple and state the aim to be 'to investigate the topographic variability.....'

Methods:
Page 4- MRI acquisition- 2 machines with different magnet strength were used. Did the authors use the exact same scanning parameters? would the difference in scanners affect results at all?

Stats- no details provided re: variables used in the regression model

Page 6, line 15- typo, ...were calculated for each.....

Results:
Page 7 line 9 typo, ... The ratio between two....

Page 7 line 13/14, 'The average length of the perimeter was 64.0 mm (SD 7 and 7.4), ranging from 46.9 to 83.7 mm'- why two SDs are presented? what are each for?

All figures need to have a legend describing what the images are, what they show etc. Currently no description provided at all. Also, labels used in images are not easily visible, suggest the authors use contrasting color for labels to improve visibility.

Editorial Office comments:

-Please supply a graphical abstract. A graphical abstract is a visual representation of the central finding or methodology of the paper. It will be displayed in the table of contents. Authors should strive to make the graphical abstract informative, interesting, visually appealing, and straightforward (no caption needed, no abbreviations except gene names). Please upload as a separate high resolution image file (tiff preferred). The technical requirements for the graphical abstracts are:
--size of the submitted image: 1200 pixels square at 300dpi
--font of the text: Arial 12-16 point (smaller fonts will not be legible online)

-Please add an Abbreviated Summary (up to 50 words) of your paper that captures the main purpose and conclusions of the work. The summary should state the main results written in the third person (‘[1st author surname] et al. report that …’) and conclusion. This summary will be used in the contents list online and should not contain any abbreviations other than those on the accepted abbreviations list and accepted gene/protein names, making it accessible to the non-specialist. Please upload it as a Word document. Allowed abbreviations: AIDS; ANOVA; AMPA; ATP; A,T,C,G; CNS; CSF; CT; DNA; ECG; EEG; EMG; GABA; HIV; MRI; NMDA; PET; PCR; RNA.

- To comply with our ethical standards, the appropriate checklist must be included (see Reporting Guideline section within 'Instructions to authors').

- Please, add a legend for each figure of the manuscript, which will include details of the figures.
