# Supplementary material for: Topographic variability of the normal circle of Willis anatomy on a paediatric population
Source: Brain Commun. 2021 Apr 3;3(2):fcab055. doi: 10.1093/braincomms/fcab055 (PMC8204365; doi:10.1093/braincomms/fcab055)
Supplement: fcab055_Supplementary_Data [file fcab055_supplementary_data.zip › Review_files.zip!/Original Submission.pdf]

OXFORD UNIVERSITY PRESS | Brain Communications

Topographic variability of the normal Willis circle anatomy on a pediatric population

Journal:	*Brain Communications*
Manuscript ID	BRAINCOM-2020-338
Manuscript Type:	Original Article
Date Submitted by the Author:	28-Oct-2020
Complete List of Authors:	ZRAFI, Wael Salem; INSERM, U 1018; Gustave Roussy; Paris-Saclay University<br>Veres, Cristina; INSERM, UMR 1018; Gustave Roussy; Paris-Saclay University; INSERM, UMR 1030<br>Dangouloff-Ros, Volodia; Assistance Publique Hôpitaux de Paris, Hôpital Necker-Enfants Malades, Pediatric radiology; Institut Imagine, INSERM U1163; Paris University; INSERM, UA 10<br>Boddaert, Nathalie; AP-HP, Hôpital universitaire Necker-Enfants malades, Paediatric Imaging; Institut Imagine, INSERM U1163; Paris University; INSERM, UA 10<br>HADDY, Nadia; INSERM, U1018; Gustave Roussy; Paris-Saclay University<br>JOURNY, Neige; INSERM, U 1018; Gustave Roussy; Paris-Saclay University<br>Allodji, Rodrigue; INSERM, U1018; Gustave Roussy; Paris-Saclay University<br>ALABDOABURAS, Mohamad; INSERM, U 1018; Gustave Roussy; Paris-Saclay University<br>DIALLO, Ibrahima; INSERM, U1018; Gustave Roussy; Paris-Saclay University; INSERM, UMR 1030<br>de Vathaire, Florent; INSERM,
Keywords:	

SCHOLARONE™ Manuscripts

Topographic variability of the normal Willis circle anatomy on a pediatric population

Wael Zrafi[1,2,3], Cristina Veres[1,2,3,4,5], Volodia Dangouloff-Ros[6,7,8,9], Nathalie Boddaert[6,7,8,9], Nadia Haddy[1,2,3], Neige Journy[1,2,3], Rodrigue Allodji[1,2,3], Mohamad Mohamad Alabdoaburas[1,2,3], Ibrahima Diallo[1,2,3,4,5]*, Florent de Vathaire[1,2,3]

[1] Radiation Epidemiology Group, Center for Research in Epidemiology and Population Health, INSERM U1018, Villejuif, F-94805, France.

[2] Gustave Roussy, Villejuif, F-94805, France.

[3] University Paris Saclay, Villejuif, F-94805, France.

[4] Molecular Radiotherapy and Innovative Therapeutics, INSERM UMR 1030, Villejuif, F-94805, France.

[5]Department of Radiation Oncology, Gustave Roussy Cancer Campus, 94800 Villejuif, France

[6] Pediatric radiology, CHU Necker Enfants Malades, Paris F-75015, France

[7] Université de Paris, Paris F-75015, France

[8] Institut Imagine UMR 1163, Paris F-75015, France

[9] INSERM Unit UA 10, Paris F-75015, France

* Corresponding author : Ibrahima Diallo,

For Review Only

Abstract

Long-term sequelae are major limitations of radiation therapy use, especially for childhood brain tumor. Circle of Willis irradiation strongly increases the long-term risk of stroke, but to establish dose-response relationship, anticipating long-term effects of new techniques, requires to perform accurate and reproducible dosimetric estimations in large cohorts of patients having received radiotherapy decades ago. For the accuracy of retrospective dose reconstruction, the topographic variability of the Circle of Willis arteries is crucial.

In order to improve retrospective dosimetry studies and dose-volume estimates to the typical Willis circles arteries, we aim to study the inter-individual topographic variability of these structures.

38 Time of flight (TOF) MRI sequences of children aged 2 to 17 years in both genders were investigated. A region growth algorithm was used for the segmentation of the cerebral arteries. A rigid registration in a common skull was performed following the anatomy of skull base foramina. The Posterior clinoid processes of the sella turcica was used as reference landmark (R0), and 5 key landmark were chosen in each segmented Willis circle , then distances between the 5 landmarks and R0 were calculated for each of the 38 subjects.

The distance between R0 and each landmark of the Willis circle followed a normal distribution, the average values ranging from 13.6 to 17.0 mm, and the standard deviations ranged from 2.6 mm to 3.0 mm, i.e. less than a fifth of the average value. The perimeter of the circle of Willis was longer in older subjects, this increase being isotropic.

Our study shows a remarkably low topographic variability the Willis circle. An important result, allowing reliable anthropomorphic phantoms-based retrospective estimations of the radiation doses delivered to these arterial structures during radiotherapy treatment

Keywords :

Circle of Willis, Population Variability, Dosimetry, Radiation therapy,

.

For Review Only

Introduction

The 5-year survival of childhood cancer patients had been significantly improved exceeding 80% nowadays in developed European countries (Merchant and Kortmann, 2018), due to multidisciplinary treatments, compliance with evidence based therapeutic protocols, new drugs and technical progress of radiotherapy allowing a better targeting of tumors and sparing of healthy tissues (Gatta *et al.*, 2014).

Thus, new public health concerns specific to long-term childhood cancer survivors, are arising and need different approaches than adult cancer survivors (Oeffinger *et al.*, 2006; Mm *et al.*, 2007; Grabow *et al.*, 2018). These health issues usually rises decades after treatments and concerns large cohorts of patients.

About 75% of pediatric cancer survivors develop at least one chronic disease during long-term follow-up such as cardiovascular, endocrine, and psychological pathologies (Oeffinger *et al.*, 2006; Mm *et al.*, 2007; Hudson *et al.*, 2013; Kralik *et al.*, 2017; Nordstrom *et al.*, 2018; Tanyildizi *et al.*, 2019; Zaorsky *et al.*, 2019).

Several studies have described the long-term iatrogenic effect of radiotherapy on arterial structures, mainly to the heart arteries, but also to the main brain arteries, showing a significant increase in morbidity and mortality (Gagliardi *et al.*, 2010; Maraldo *et al.*, 2012; Darby *et al.*, 2013; Kralik *et al.*, 2017; Nordstrom *et al.*, 2018; Tanyildizi *et al.*, 2019; Zaorsky *et al.*, 2019).

Haddy et al. had investigated 23 deaths due to cerebrovascular diseases among 4227 childhood cancer survivors, showing a 22% increase in cerebrovascular mortality for each Gray received in the prepontine cistern (Haddy *et al.*, 2011).

Similarly, El-Fayech et al. study showed an 8.5 fold increase in stroke risk for patients who received radiation therapy. In this study, the dose received by the Willis circle appears to be the best predictor of stroke risk (El-Fayech *et al.*, 2017).

Another study with vessel wall imaging MRI showed a vascular wall changes to the internal carotid artery and to the basilar circulation after radiochemotherapy occurring in 64 % of exposed children but failed to link these changes to the delivered radiation dose(Tanyildizi *et al.*, 2019).

Despite, the rising awareness about the radiation related toxicity, recommendation for the contouring and dose constraints to the Willis circle are yet lacking.

For Review Only

Our aim was to investigate the retrospective dose estimation reliability, through the assessment of the topographic variability of the typical Willis circle arteries in a pediatric population, in order to improve the methods used for doses-volume estimates to the Willis circle arteries in retrospective dosimetric studies.

Methods

Study population:

We have investigated 38 consecutive Time of Flight (TOF) MRI sequences of in children aged from 2 to 17 years with no anomalies nor anatomic variant of the Willis circle, as confirmed by a senior pediatric neuroradiologist (VDR, 9 years of experience). There were 19 boys and 19 girls, with a median age of 8 years ranging from 2 to 17 (table S-I). Most of them were consulting for headaches in context of sickle cell diseases, without evidence of vascular abnormality on MRI.

All MRI were performed in same Radiology department, two machine were used the DISCOVERY MR750 and the Optima MR450w, 3 and 1.5 Tesla, The slice thickness was 1.2 mm, and the reconstruction matrix size was 512 X 512 pixels for most of them.

Image processing:

Brain artery segmentation was performed using a region growing algorithm (Osirix® software). The segmentation was focused on Willis' circle, as being reported to be the main predictor of stroke risk (Supplementary Fig. 1) (12).

ISOgray™ (Dosisoft, Cachan, France) software was used to import the sequences obtained by OsiriX, and manually label each of the Willis circle arteries.

In order to ensure the reproducibility of the labelling of the contoured arterial structures, we follow the following labeling process:

1) we start from below, by the first slide showing the transit of arteries by the base of skull foramina. in this slide three arterial structures are identified:

- the left and right internal carotid arteries (ICA), each one passes through its respective carotid canal.
- the basilar artery (BA) passes through the Magnum Foramen close to the anterior surface of the brain stem.

For Review Only

2) These structures were segmented until the bifurcation of each one of them:

- bifurcation of the BA into left and right posterior cerebral arteries (PCA),
- bifurcation of each ICA into left and right middle cerebral arteries (MCA), left and right anterior cerebral arteries (ACA).

3) These arteries were segmented until their first bifurcation, in order to avoid segmentation of the distal arteries

4) The communicating arteries were segmented as following:
- the anterior communicating artery (ACoA), as being median arterial segment that connects the left and right ACA.
- the left and right posterior communicating arteries (ACoP), which connects respectively each ICA to the corresponding PCA

Thus, 12 structures were segmented for each MRI (38 MRIs)

Fig. 1 shows an example of some steps and the result obtained with the above segmentation process.

Registration:

In order to investigate the topographic variability between the segmented Willis' circle of 38 subjects, these volumes were rigidly registered in the same 3D space and a common coordinates system. We proceeded by a matching the segmented Willis circles to a skull bone imaging respecting the different anatomical landmarks, in particular the base of skull foramina of the left and right ICA in the skull base.

This technique allows to adjust and take into account the rotational and translational differences in position of the head when performing the MRI, and to obtain a good registration of the different Willis circles.

Variability investigations:

To study the topographic variability of the Willis Polygon, we defined 5 easily identifiable anatomical landmarks in each Willis circle.

L1: bifurcation of the right ICA in right ACA and MCA.

L2: bifurcation of the left ICA in left ACA and MCA.

L3: top of the right cavernous sinus

For Review Only

L4: top of the left cavernous sinus

L5: bifurcation of the BA in left and right PCA

Thus, we obtained The X, Y, Z coordinates of each landmark. Similarly, we defined R0, as being the apex of the posterior lip of the turcica sella as a common reference landmark (Fig. 2). The distances between R0 and the landmarks L1 to L5, as well as the distances between landmarks, and the perimeter L1-L2-L5, considered as a surrogate of the Willis circle circumference (Fig. 2 and Supplementary Fig2) were calculated or each of the 38 subjects.

Statistical methods:

Quantile to Quantile plots (Q-Q plots) and Shapiro-Wilk test were used to investigate the normality of the distribution of the different calculated distances(Wilk and Gnanadesikan, 1968).

In addition to the mean and standard deviation, the coefficient of variation of each distance was calculated to describe the relative dispersion of these measurements(Brown, 1998).

Generalized linear models were used to investigate the variability and the predictors of distances between the different landmarks(Graybill, 1976).

We studied the variability of distances between the skull base and the five landmarks as first step, then the variability of distances within the Willis circle, we calculated the ratio L1-L3 / L2-L4 as an indicator of the symmetry (as they measure the same distance in the right and left internal carotid arteries).

Data availability statement:

Age, weight, X, Y, and Z coordinates of the 5 landmarks for the 38 subject data are available in the supplementary material.

Further data used in this work will be available upon reasonable request to the corresponding author

Results:

Distances from R0 to any of the L1-L5 landmarks ranged from 7.0 to 24.4 mm (table I). No significant deviance from normality was observed in the distribution of these 5 distances, as shown by Q-Q plots (Supplementary Fig. 3) and the Shapiro-Wilk test. When considering both gender together, the variability was remarkably low, the coefficients of variation ranging from 0.15 to 0.22, the findings remaining similar when considering each gender separately. Overall,

For Review Only

L1, L2, L3 and L4 were almost equidistant from R0, L5 being lightly (15% lesser than the average distance) nearest for R0 than the mean of the four other landmarks (16.1mm).

Distances between 2 landmarks within the Willis polygon have a remarkably low variation, the coefficients of variation ranging from 0.12 to 0.18. The ratio between tow symmetrical distances, here L1-L3 and L2-L4 was consistently close to one, and the (L1-L2-L5) perimeter expresses a low variability, it's coefficient of variation being the lowest. The average length of the perimeter was 64.0 mm (SD 7 and 7.4), ranging from 46.9 to 83.7 mm.

In a multivariate model, Willis' circle circumference surrogate (L1-L2-L5) was significantly linked to age, the linear regression coefficient was 0.62 (SD = 0.21, p= 0.005), but not influenced by gender (p=0.9) (Fig. 4). This increase in the perimeter for age increasing, was most important in the antero-posterior axis (p=0.02 and 0.001, respectively for L1-L5 and L2-5) than in the lateral axis (L1-L2, p=0.2) (Table 2, Supplementary Fig. 3). In this model, no significant deviance from normality was observed in the distribution of residuals, as shown in (Fig. 4).

For Review Only

Discussion:

Our study showed a low variability for the constituent arteries of Willis circle, as the distance between each of the 5 landmarks chosen to represent the shape of this structure. The Willis circle grows in an isotropic way, the variation coefficients being very low range 0.10 - 0.17. When considering the distance with the bone landmark the variation coefficients were slightly higher 0.15 - 0.22, but remained globally low. As a general matter, these distances followed a normal distribution.

In a linear regression, a significant increase in the Willis circle perimeter of 0.62 mm in average for each year was observed, which has to be correlated to the natural growth of the skull bones, since the development of the cerebral arteries, especially the internal carotid arteries, seems closely related to that of the skull bones. We did not observe significant effect of gender. Nevertheless, the small number of patients did not permit us to deeply investigate the shape of the relation between gender, age and Willis circle parameters.

The data published by the World Health Organization (WHO) on cranial perimeter (CP) shows that most of the growth takes place between birth and 2 years, the average CP increasing from 339 - 345 to 472 - 482 mm. After 2 years the growth rate is significantly lower and reaches 499 - 507 mm at 5 years (De Onis, 2007), period during which the average annual increase of the

median CP is of 1.6% of its value (De Onis, 2007). In another study, performed by NIH study, a linear growth of the total brain volume for males and a quadratic growth for females in a population of 4 to 18 years, the total brain volume globally increasing by 2.03% per year, i.e. an increase of 1.27 % brain perimeter ('Total and Regional Brain Volumes in a Population-Based Normative Sample from 4 to 18 Years', 2012). This last value is similar to the annual increase of about 1% (0,6m/60mm) we observed in our sample in which age ranged from 2 to 18.

In this work, the registration of the Willis Polygon in the coordinate system centered on the skull bone foramina, makes our results independent of the position of the child head and accounts for the possible movement of rotation, flexion and extension that are frequent during the MRI procedure, which can vary according to the position during radiotherapy treatment. On the other hand, the registration as well as identification of the 5 landmarks on each one of the 38 MRI are done manually and are therefore subject to the subjectivity of the operator, an inconvenient that could be bypassed with the development of automatic and semi-automatic registration and contouring methods (Pekar *et al.*, 2004). Additionally, our work was limited to 5 anatomically and easily recognizable key landmarks, and could be improved by including most of the Willis circle shape and pixels distributed over the entire volume of Willis circle. In this work, we were only interested in the topographic variability of the Willis circle constituent arteries. Hence we included only MRIs with no anomalies or anatomical variant, as we are not interested on the morphological variability of the Willis Polygon largely described in the literature. Lastly most subjects in our sample had an MRI motivated by the suspicion of a stroke in a context of sickle cell disease. Up to now, no study has shown specific anatomical characteristic of the Willis circle in these patients (Alshehri *et al.*, 2020).

In our knowledge this is the only study analyzing the topographic variability of the Willis Polygon. It was motivated by reports of long-term cerebrovascular toxicity in the adulthood of pediatric cancer survivors, based on retrospective dosimetry, which showed a correlation between doses delivered to Willis' Polygon and long-term cerebrovascular toxicities (11,12). This study shows that in anthropomorphic phantoms used for retrospective dose-reconstruction, the Willis's arterial circle can be represented by a mean shape derived from segmented MRIs, this process being feasible and reliable.

For Review Only

Funding:

W.Z received support as recipient of the DUERTECC/EURONCO education grant (Diplome Universitaire Européen de Recherche Translationelle et Clinique en Cancerologie – no grant number applicable) and by the Foundation ARC for Cancer Resarch (grant no. Pop-HaRC 201401208).

The funding source had no role in the preparation of this article.

Competing interests:

The authors report no competing interests.

For Review Only

References

Alshehri E, Dmytriw AA, Chavhan GB, Amirabadi A, Shroff MM, Williams S, et al. The role of MRA in pediatric sickle cell disease with normal transcranial Doppler imaging velocities. J Stroke Cerebrovasc Dis Off J Natl Stroke Assoc 2020; 29: 104864.

Brown CE. Coefficient of Variation. In: Brown CE, editor(s). Applied Multivariate Statistics in Geohydrology and Related Sciences. Berlin, Heidelberg: Springer; 1998. p. 155–7

Darby SC, Ewertz M, McGale P, Bennet AM, Blom-Goldman U, Brønnum D, et al. Risk of ischemic heart disease in women after radiotherapy for breast cancer. N Engl J Med 2013; 368: 987–98.

De Onis M. WHO child growth standards: head circumference-for-age, arm circumference-for-age, triceps skinfold-for-age and subscapular skinfold-for-age: methods and development. World Health Organization; 2007

El-Fayech C, Haddy N, Allodji RS, Veres C, Diop F, Kahlouche A, et al. Cerebrovascular Diseases in Childhood Cancer Survivors: Role of the Radiation Dose to Willis Circle Arteries. Int J Radiat Oncol Biol Phys 2017; 97: 278–86.

Gagliardi G, Constine LS, Moiseenko V, Correa C, Pierce LJ, Allen AM, et al. Radiation Dose–Volume Effects in the Heart. Int J Radiat Oncol Biol Phys 2010; 76: S77–85.

Gatta G, Botta L, Rossi S, Aareleid T, Bielska-Lasota M, Clavel J, et al. Childhood cancer survival in Europe 1999–2007: results of EUROCARE-5—a population-based study. Lancet Oncol 2014; 15: 35–47.

Grabow D, Kaiser M, Hjorth L, Byrne J, Alessi D, Allodji RS, et al. The PanCareSurFup cohort of 83,333 five-year survivors of childhood cancer: a cohort from 12 European countries. Eur J Epidemiol 2018; 33: 335–49.

Graybill FA. Theory and application of the linear model. Duxbury press North Scituate, MA; 1976

Haddy N, Mousannif A, Tukenova M, Guibout C, Grill J, Dhermain F, et al. Relationship between the brain radiation dose for the treatment of childhood cancer and the risk of long-term cerebrovascular mortality. Brain J Neurol 2011; 134: 1362–72.

Hudson MM, Ness KK, Gurney JG, Mulrooney DA, Chemaitilly W, Krull KR, et al. Clinical Ascertainment of Health Outcomes Among Adults Treated for Childhood Cancer. JAMA 2013; 309: 2371–81.

Kralik SF, Watson GA, Shih C-S, Ho CY, Finke W, Buchsbaum J. Radiation-Induced Large Vessel Cerebral Vasculopathy in Pediatric Patients With Brain Tumors Treated With Proton Radiation Therapy. Int J Radiat Oncol Biol Phys 2017; 99: 817–24.

Maraldo MV, Brodin NP, Vogelius IR, Aznar MC, Munck Af Rosenschöld P, Petersen PM, et al. Risk of developing cardiovascular disease after involved node radiotherapy versus mantle field for Hodgkin lymphoma. Int J Radiat Oncol Biol Phys 2012; 83: 1232–7.

For Review Only

Mm G, Mc C-U, Lc K, C van den B, Hj van der P, Rc H, et al. Medical assessment of adverse health outcomes in long-term survivors of childhood cancer. JAMA 2007; 297: 2705–15.

Nordstrom M, Felton E, Sear K, Tamrazi B, Torkildson J, Gauvain K, et al. Large Vessel Arteriopathy After Cranial Radiation Therapy in Pediatric Brain Tumor Survivors. J Child Neurol 2018; 33: 359–66.

Oeffinger KC, Mertens AC, Sklar CA, Kawashima T, Hudson MM, Meadows AT, et al. Chronic Health Conditions in Adult Survivors of Childhood Cancer. N Engl J Med 2006; 355: 1572–82.

Pekar V, McNutt TR, Kaus MR. Automated model-based organ delineation for radiotherapy planning in prostatic region. Int J Radiat Oncol Biol Phys 2004; 60: 973–80.

Tanyildizi Y, Keweloh S, Neu MA, Russo A, Wingerter A, Weyer-Elberich V, et al. Radiation-induced vascular changes in the intracranial irradiation field in medulloblastoma survivors: An MRI study. Radiother Oncol J Eur Soc Ther Radiol Oncol 2019; 136: 50–5.

Wilk MB, Gnanadesikan R. Probability plotting methods for the analysis of data. Biometrika 1968; 55: 1–17.

Zaorsky NG, Zhang Y, Tchelebi LT, Mackley HB, Chinchilli VM, Zacharia BE. Stroke among cancer patients. Nat Commun 2019; 10: 5172.

Total and Regional Brain Volumes in a Population-Based Normative Sample from 4 to 18 Years: The NIH MRI Study of Normal Brain Development. Cereb Cortex 2012; 22: 1–12.

Pediatric Radiation Oncology. Springer International Publishing; 2018

For Review Only

Table 1:

Distribution parameters of distances between landmarks in 38 subjects.

	Mean	Median	Standard-deviation	Coefficient of variation	Range	Shapiro test for normality
Whole sample (n=38)						
R0-L1	15.5	15.4	2.7	0.17	10.7 - 21.9	0.60
R0-L2	16.7	16.0	2.9	0.17	11.1 - 22.4	0.27
R0-L3	15.3	14.8	2.8	0.18	10.6 - 20.9	0.33
R0-L4	17.0	16.7	2.6	0.15	11.5 - 24.4	0.41
R0-L5	13.6	13.6	3.0	0.22	7.0 - 19.6	0.88
L1-L2	26.8	25.9	3.4	0.13	21.4-36.8	0.03
L1-L3	16.4	16.3	2.5	0.16	12.2-22.1	0.41
L1-L5	18.8	18.8	2.7	0.14	12.8-23.3	0.46
L2-L4	15.8	15.6	2.3	0.14	11.7-22.4	0.21
L2-L5	18.4	18.3	2.8	0.15	12.8-24.1	0.47
(L1-L3)/(L2-L4)	1.04	1.03	0.1	0.10	0.80-1.2	0.82
L1-L2-L5	64.0	63.5	7.1	0.11	46.9-83.7	0.81
Females (n=19)						
R0-L1*	14.4	14.3	2.3	0.16	10.7 - 21.4	0.05
R0-L2	16.5	15.8	2.9	0.18	12.2 - 22.4	0.05
R0-L3	15.2	14.7	2.5	0.16	11.4 - 20.9	0.55
R0-L4	17.2	16.3	3.0	0.17	13.0 – 14.4	0.24
R0-L5	13.6	14.3	2.7	0.20	8.0 – 18.0	0.83
L1-L2	26.8	26.0	3.6	0.14	22.0-36.8	0.04
L1-L3	16.0	15.2	2.9	0.18	12.2-21.2	0.13
L1-L5	18.8	18.1	2.5	0.13	15.1-23.3	0.07
L2-L4	15.4	15.0	2.5	0.16	11.7-20.7	0.44
L2-L5	18.6	18.1	3.1	0.17	13.5-24.1	0.24

For Review Only

(L1-L3)/(L2-LP4)	1.05	1.03	0.12	0.11	0.8-1.2	0.47
L1-L2-L5	64.2	64.0	7.4	0.12	54.0-83.7	0.25
Males (n=19)						
R0-L1*	16.5	16.7	2.6	0.16	12.0 - 21.9	0.81
R0-L2	17.0	17.0	2.8	0.16	11.1 – 22.3	1
R0-L3	15.3	14.9	3.1	0.20	10.6 – 20.3	0.18
R0-L4	16.8	16.7	2.2	0.13	11.5 – 20.3	0.42
R0-L5	13.6	13.5	3.4	0.25	7.0 – 19.6	0.76
L1-L2	26.8	25.7	3.3	0.12	21.4-32.5	0.29
L1-L3	16.8	17.0	2.1	0.13	13.4-22.1	0.46
L1-L5	18.7	19.1	2.9	0.15	12.8-23.2	0.81
L2-L4	16.3	16.1	2.0	0.12	13.4-22.4	0.03
L2-L5	18.2	18.5	2.7	0.15	12.8-22.3	0.66
(L1-L3)/(L2-L4)	1.04	1.04	0.1	0.09	0.9-1.2	0.88
L1-L2-L5	63.7	63.1	7.0	0.11	46.9-76.1	0.78

R0 : Posterior clinoid processes of the sella turcica

L1: intersection of right internal carotid artery and middle and anterior cerebral artery

L2: intersection of left internal carotid artery and middle and anterior cerebral artery

L3: top of the right cavernous sinus

L4: top of the left cavernous sinus

L5: end of basilar artery and birth of the right and left posterior cerebral arteries.

* p-value for difference between genders = 0.01

For Review Only

Table 2:

Role of age and gender in the variance of distance between some key landmarks in Circle Willis in 38 subjects.

	Age			Gender			p-value for interaction between age and gender
	Coefficient	SD	p-value	Coefficient	SD	p-value	
R0-L1	0.094	0.078	0.2	-2.20	0.79	0.009	0.6
R0-L2	0.073	0.094	0.4	-0.51	0.94	0.6	0.9
R0-L3	0.12	0.091	0.2	-0.18	0.92	0.8	0.06
R0-L4	0.083	0.086	0.3	0.41	0.84	0.6	0.2
R0-L5	-0.016	0.099	0.9	0.0025	1.01	0.9	0.6
L1-L3	-0.017	0.083	0.9	-0.74	0.84	0.4	0.1
L2-L4	0.0097	0.075	0.9	-0.86	0.75	0.3	0.2
(L1-L3)/(L2-L4)	-0.00052	0.0034	0.9	0.0098	0.034	0.8	0.3
L1-L2-L5	0.62	0.21	0.005	0.18	2.12	0.9	0.3
L1-L2	0.15	0 11	0.2	-0.084	1.11	0.9	0.4
L1-L5	0.27	0.076	0.001	-0.026	0.77	0.9	0.6
L2-L5	0.20	0.087	0.02	0.29	0.88	0.7	0.2

R0 : Posterior clinoid processes of the sella turcica

L1: intersection of right internal carotid artery and middle and anterior cerebral artery

L2: intersection of left internal carotid artery and middle and anterior cerebral artery

L3: top of the right cavernous sinus

L4: top of the left cavernous sinus

L5: end of basilar artery and birth of the right and left posterior cerebral arteries.

* p-value for difference between genders = 0.01

For Review Only

Figure 1:

Contouring and labelling process.

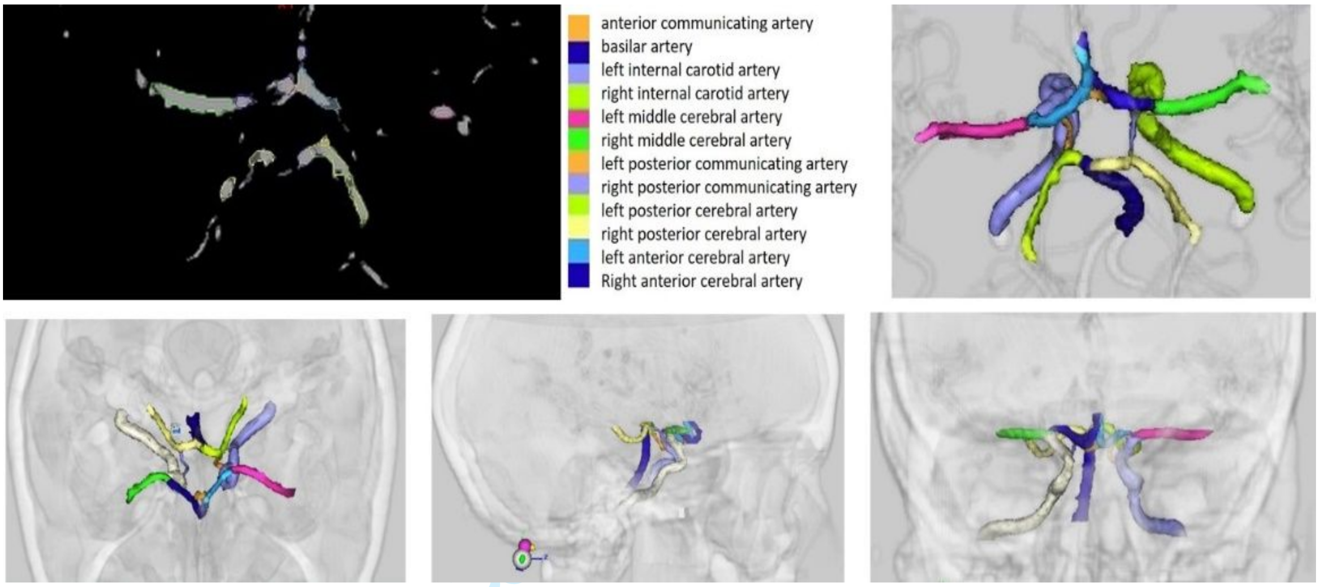

review Only

Figure 2

3D representation of the segmented Willis Circle and the skull base.

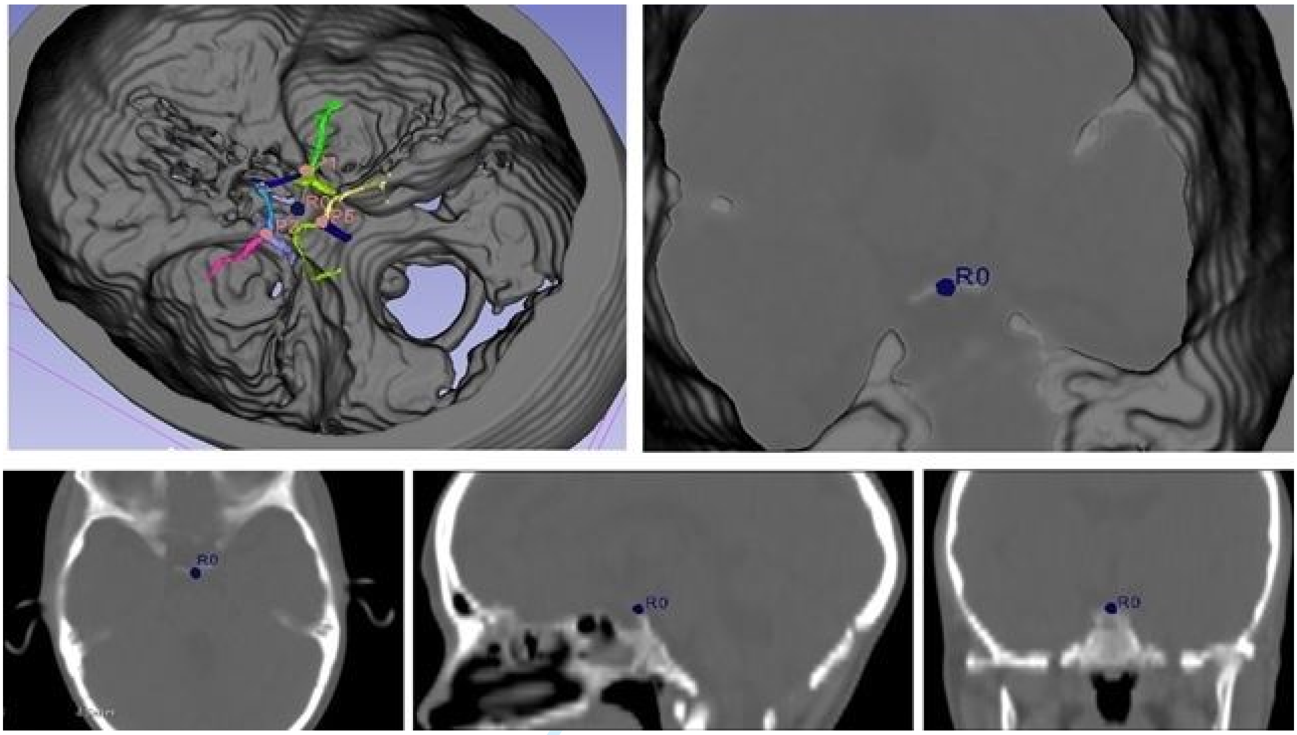

view Only

Figure 3:

Willis perimeter growth according to age.

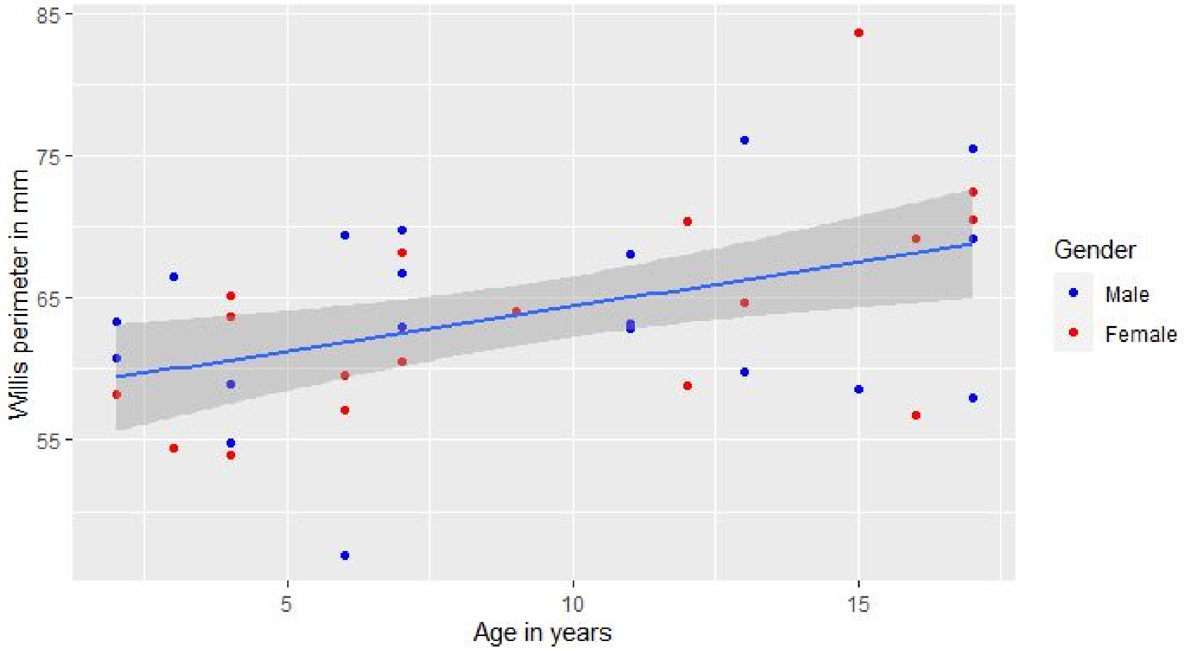

For Peer Review Only

Figure 4:

Residual L1 L2 L5 distances distribution.

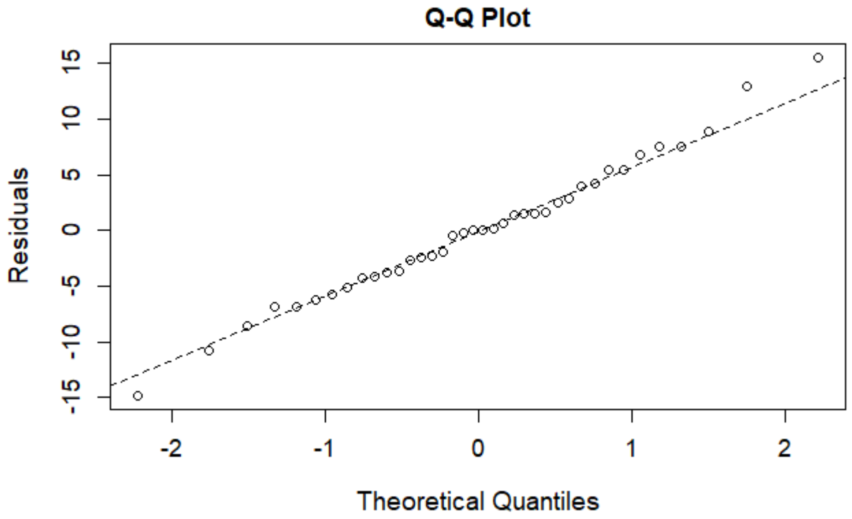

r Review Only

Supplementary material

For Review Only

Supplementary Table 1:

Topographic coordinates of posterior clinoid processes of sella turcica and of 5 topographic landmarks of the Circle of Willis.

Patient	Age (year)	Weight	Gender	Points	X	Y	Z
0				R0	1,4731	-10,6774	-106,193
1	3	11	female	L1	12,4611	-8,67756	-102,596
1	3	11	female	L2	-9,4761	-8,07325	-101,372
1	3	11	female	L3	12,0857	-1,74287	-112,591
1	3	11	female	L4	-8,38509	-0,19162	-110,187
1	3	11	female	L5	0,071609	-20,1301	-103,502
2	4	15	female	L1	10,4379	-10,4373	-95,5877
2	4	15	female	L2	-13,6344	-11,6794	-99,4575
2	4	15	female	L3	9,73716	-1,41712	-110,866
2	4	15	female	L4	-12,535	-3,25719	-111,838
2	4	15	female	L5	-3,05978	-19,1669	-95,6015
3	2	13	female	L1	11,3272	-14,5215	-104,535
3	2	13	female	L2	-13,0524	-12,9634	-105,975
3	2	13	female	L3	9,65831	-5,36062	-116,618
3	2	13	female	L4	-12,3915	-2,67734	-117,003
3	2	13	female	L5	-0,3788	-25,2685	-106,981
4	4	21	female	L1	16,5266	-15,89	-102,775
4	4	21	female	L2	-12,3088	-14,4512	-99,5532
4	4	21	female	L3	12,2377	-6,49468	-111,409
4	4	21	female	L4	-10,7395	-3,63181	-111,632

4	4	21	female	L5	-0,21895	-25,5306	-102,986
5	4	22	female	L1	10,4815	-9,46248	-94,8458
5	4	22	female	L2	-17,9173	-8,87284	-98,5418
5	4	22	female	L3	11,1829	1,55192	-112,335
5	4	22	female	L4	-13,5806	2,41263	-114,742
5	4	22	female	L5	-5,91314	-18,7189	-93,8474
6	2	12	male	L1	16,5663	-0,93451	-101,864
6	2	12	male	L2	-8,67082	-1,86393	-101,744
6	2	12	male	L3	11,8953	2,44146	-115,07
6	2	12	male	L4	-5,99259	3,70634	-115,51
6	2	12	male	L5	1,39393	-13,645	-99,9005
7	2	17	male	L1	16,1692	-14,9126	-96,4265
7	2	17	male	L2	-13,3973	-14,0654	-98,0901
7	2	17	male	L3	8,73166	-3,83917	-110,947
7	2	17	male	L4	-9,79556	-2,92088	-111,056
7	2	17	male	L5	0,812344	-22,2171	-99,1878
8	4	20	male	L1	11,2971	-9,49798	-97,5886
8	4	20	male	L2	-13,4163	-9,0802	-98,1076
8	4	20	male	L3	7,55061	-0,37726	-108,736
8	4	20	male	L4	-9,50457	1,19403	-109,435
8	4	20	male	L5	-0,55296	-20,7348	-100,684
9	4	13	male	L1	14,9532	-8,5622	-98,1334
9	4	13	male	L2	-8,41787	-7,78057	-97,7033
9	4	13	male	L3	10,4185	0,903376	-112,951

9	4	13	male	L4	-9,0324	1,3582	-111,62
9	4	13	male	L5	3,23425	-18,5391	-96,1698
10	3	12	male	L1	14,8803	-14,1214	-99,2049
10	3	12	male	L2	-11,8881	-13,7119	-98,9355
10	3	12	male	L3	15,786	-3,62655	-113,453
10	3	12	male	L4	-10,4861	-2,38352	-111,786
10	3	12	male	L5	-0,74834	-27,9822	-102,813
11	9	30	female	L1	12,8922	-13,7242	-98,1149
11	9	30	female	L2	-11,5548	-12,1844	-100,112
11	9	30	female	L3	13,1552	-2,84764	-110,101
11	9	30	female	L4	-8,143	-2,96099	-110,466
11	9	30	female	L5	3,48646	-28,0298	-101,7
12	6	20	female	L1	13,8902	-12,8769	-97,7206
12	6	20	female	L2	-12,4082	-14,0894	-99,2715
12	6	20	female	L3	10,4658	-3,58898	-110,095
12	6	20	female	L4	-11,3923	-3,34161	-109,543
12	6	20	female	L5	0,145353	-23,578	-99,0609
13	7	40	female	L1	13,8458	-12,6645	-96,4142
13	7	40	female	L2	-17,2258	-14,6689	-99,2421
13	7	40	female	L3	14,5307	2,82049	-110,906
13	7	40	female	L4	-12,2033	-0,358	-108,603
13	7	40	female	L5	3,25596	-22,1541	-101,334
14	7	20	female	L1	10,7562	-8,523	-99,9643
14	7	20	female	L2	-13,5188	-8,71129	-101,461

14	7	20	female	L3	7,78507	-1,18571	-109,623
14	7	20	female	L4	-10,2062	-0,07482	-109,817
14	7	20	female	L5	-0,9047	-20,1495	-107,466
15	6	17	female	L1	12,3069	-7,01952	-100,455
15	6	17	female	L2	-11,5295	-7,03353	-100,139
15	6	17	female	L3	6,95148	-1,70688	-110,689
15	6	17	female	L4	-7,24142	-0,46602	-108,84
15	6	17	female	L5	-1,11154	-17,9687	-104,055
16	6	25	male	L1	8,45082	-25,4899	-109,582
16	6	25	male	L2	-12,7845	-23,3449	-109,901
16	6	25	male	L3	8,79037	-10,5268	-114,299
16	6	25	male	L4	-11,1615	-8,30757	-115,533
16	6	25	male	L5	-2,34377	-26,9905	-116,286
17	7	30	male	L1	16,32	-14,7663	-90,6178
17	7	30	male	L2	-15,9952	-12,4841	-92,474
17	7	30	male	L3	13,3351	0,560729	-106,298
17	7	30	male	L4	-9,81358	1,28643	-109,039
17	7	30	male	L5	3,50149	-18,9074	-93,2792
18	7	30	male	L1	18,5597	-10,1554	-102,002
18	7	30	male	L2	-10,2638	-5,66167	-101,569
18	7	30	male	L3	15,1085	-1,42662	-115,748
18	7	30	male	L4	-9,04839	-0,23867	-113,933
18	7	30	male	L5	1,24095	-21,6891	-102,158
19	6	25	male	L1	13,6607	-14,6402	-93,6982

19	6	25	male	L2	-14,4562	-9,01759	-94,2528
19	6	25	male	L3	9,99085	-3,9329	-106,4
19	6	25	male	L4	-11,0892	-1,22843	-105,526
19	6	25	male	L5	-2,04525	-25,1719	-99,3005
20	7	27	male	L1	11,6155	-12,868	-100,205
20	7	27	male	L2	-11,9821	-12,8107	-99,0316
20	7	27	male	L3	13,6194	-0,52261	-113,089
20	7	27	male	L4	-10,5581	0,347169	-111,234
20	7	27	male	L5	0,621596	-28,4947	-100,448
21	11	37	female	L1	12,2218	-6,33031	-99,6469
21	11	37	female	L2	-11,3287	-6,89661	-99,182
21	11	37	female	L3	10,2265	0,176999	-113,016
21	11	37	female	L4	-11,7244	0,429114	-111,737
21	11	37	female	L5	-1,45091	-20,8383	-111,72
22	12	42	female	L1	16,0012	-10,0331	-101,488
22	12	42	female	L2	-8,43346	-10,6759	-97,6692
22	12	42	female	L3	15,0907	-2,80098	-112,697
22	12	42	female	L4	-11,872	-3,7765	-112,556
22	12	42	female	L5	2,37931	-22,7905	-114,135
23	12	33	female	L1	14,1756	-17,1742	-104,944
23	12	33	female	L2	-11,8224	-16,3899	-106,798
23	12	33	female	L3	13,5466	-6,03942	-113,161
23	12	33	female	L4	-11,6603	-4,58137	-113,617
23	12	33	female	L5	0,418225	-25,3136	-110,822

24	13	45	female	L1	15,6681	-11,0593	-101,192
24	13	45	female	L2	-10,2376	-12,938	-99,3815
24	13	45	female	L3	11,2889	-0,90619	-110,998
24	13	45	female	L4	-7,9117	-1,75101	-110,092
24	13	45	female	L5	2,05699	-22,5619	-109,883
25	11	45	male	L1	12,2792	-11,871	-100,127
25	11	45	male	L2	-13,4468	-11,259	-100,24
25	11	45	male	L3	14,8664	-0,22876	-113,883
25	11	45	male	L4	-14,1151	-0,10041	-113,561
25	11	45	male	L5	-0,10856	-24,2217	-105,069
26	11	42	male	L1	12,2272	-0,01931	-94,3461
26	11	42	male	L2	-12,8347	-2,10842	-95,2629
26	11	42	male	L3	9,88648	5,42605	-109,619
26	11	42	male	L4	-10,6814	5,37959	-108,491
26	11	42	male	L5	-1,01651	-14,9874	-92,9493
27	13	89	male	L1	15,9122	-6,34557	-102,277
27	13	89	male	L2	-15,7173	-8,336	-102,075
27	13	89	male	L3	16,7984	-1,21463	-115,517
27	13	89	male	L4	-14,2689	-1,48453	-115,204
27	13	89	male	L5	1,03302	-20,9149	-109,653
28	13	30	male	L1	8,83686	-7,15242	-95,6225
28	13	30	male	L2	-15,365	-7,6861	-99,1914
28	13	30	male	L3	9,75869	0,456016	-111,182
28	13	30	male	L4	-11,9002	0,787975	-111,702
28	13	30	male	L5	-1,74524	-17,914	-104,424

29	11	50	male	L1	14,3662	-17,1569	-99,1108
29	11	50	male	L2	-16,7726	-14,7137	-96,9141
29	11	50	male	L3	13,113	-3,85444	-110,078
29	11	50	male	L4	-13,7299	-2,92599	-108,295
29	11	50	male	L5	0,33128	-24,4075	-102,846
30	16		female	L1	9,70528	-17,2785	-99,3566
30	16		female	L2	-13,4891	-16,6364	-100,735
30	16		female	L3	13,039	-3,58782	-112,684
30	16		female	L4	-15,089	-1,92711	-115,265
30	16		female	L5	-2,88129	-27,4738	-105,933
31	17		female	L1	13,7721	-14,0602	-96,4418
31	17		female	L2	-15,8055	-12,9235	-96,8083
31	17		female	L3	13,4924	-1,42203	-113,23
31	17		female	L4	-11,3351	-1,69169	-110,191
31	17		female	L5	0,290393	-26,394	-102,293
32	15		female	L1	19,2308	-10,8792	-94,2257
32	15		female	L2	-17,4618	-7,80777	-94,6296
32	15		female	L3	11,6256	-0,8792	-104,972
32	15		female	L4	-12,1449	0,21033	-104,116
32	15		female	L5	0,770037	-21,5979	-102,156
33	16		female	L1	12,1806	-7,96198	-98,5876
33	16		female	L2	-18,3021	-6,65085	-100,672
33	16		female	L3	15,8164	3,10843	-112,62
33	16		female	L4	-16,3198	4,89413	-112,015

33	16	female	L5	-3,92461	-18,9162	-98,2435
34	17	female	L1	15,1491	-7,12433	-101,189
34	17	female	L2	-13,4767	-7,99102	-104,112
34	17	female	L3	14,043	-0,00352	-114,075
34	17	female	L4	-14,3719	0,131365	-114,971
34	17	female	L5	-0,20541	-23,1482	-107,875
35	17	male	L1	15,4687	-8,63135	-96,7132
35	17	male	L2	-7,4652	-11,8582	-99,6487
35	17	male	L3	11,7505	-2,03349	-112,642
35	17	male	L4	-9,28248	-1,29147	-112,365
35	17	male	L5	0,978605	-21,8826	-103,853
36	17	male	L1	19,3232	-10,9375	-97,2527
36	17	male	L2	-11,2255	-9,21615	-96,912
36	17	male	L3	12,2929	-0,91649	-104,828
36	17	male	L4	-7,36763	0,10535	-105,681
36	17	male	L5	2,0502	-21,945	-108,238
37	15	male	L1	12,2025	-16,8313	-95,0128
37	15	male	L2	-11,9207	-18,2667	-97,3011
37	15	male	L3	11,2735	-6,74366	-106,932
37	15	male	L4	-8,37241	-4,89422	-107,829
37	15	male	L5	-0,96798	-27,8456	-102,579
38	17	male	L1	15,2463	-10,9042	-96,5168
38	17	male	L2	-11,8437	-12,1689	-96,3893
38	17	male	L3	12,6668	-1,46475	-105,61

38	17	male	L4	-12,4008	-0,3176	-105,928
38	17	male	L5	-0,34799	-23,2794	-107,338

For Review Only

Supplementary Figure 1

3D representation of the result after automated growing region segmentation

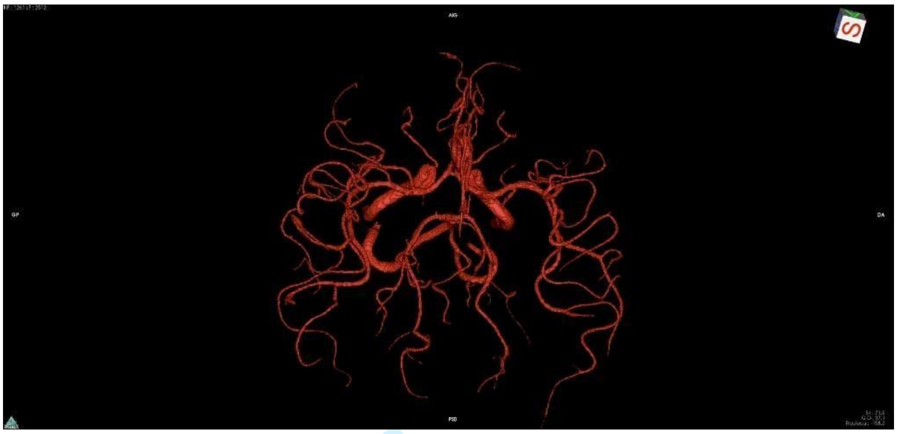

For Review Only

Supplementary Figure 2:

3D representation of the segmented Willis circle

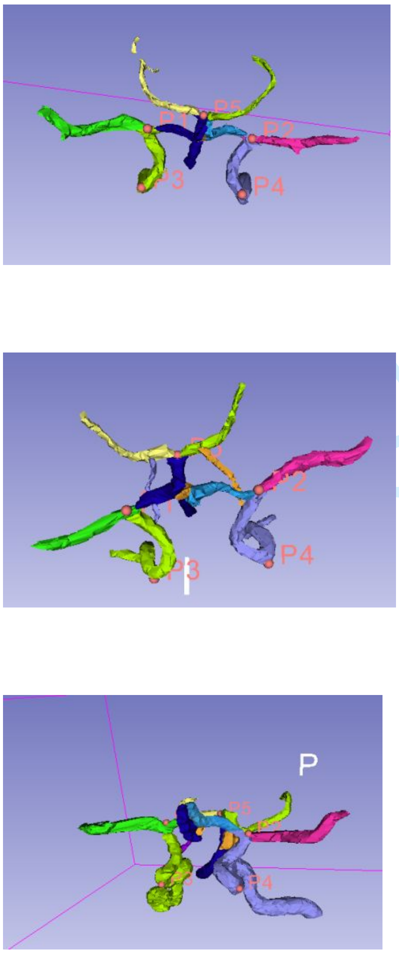

For Review Only

Supplementary Figure 3:

Q-Q plots of the distances between the reference landmark and the five circle of Willis landmarks

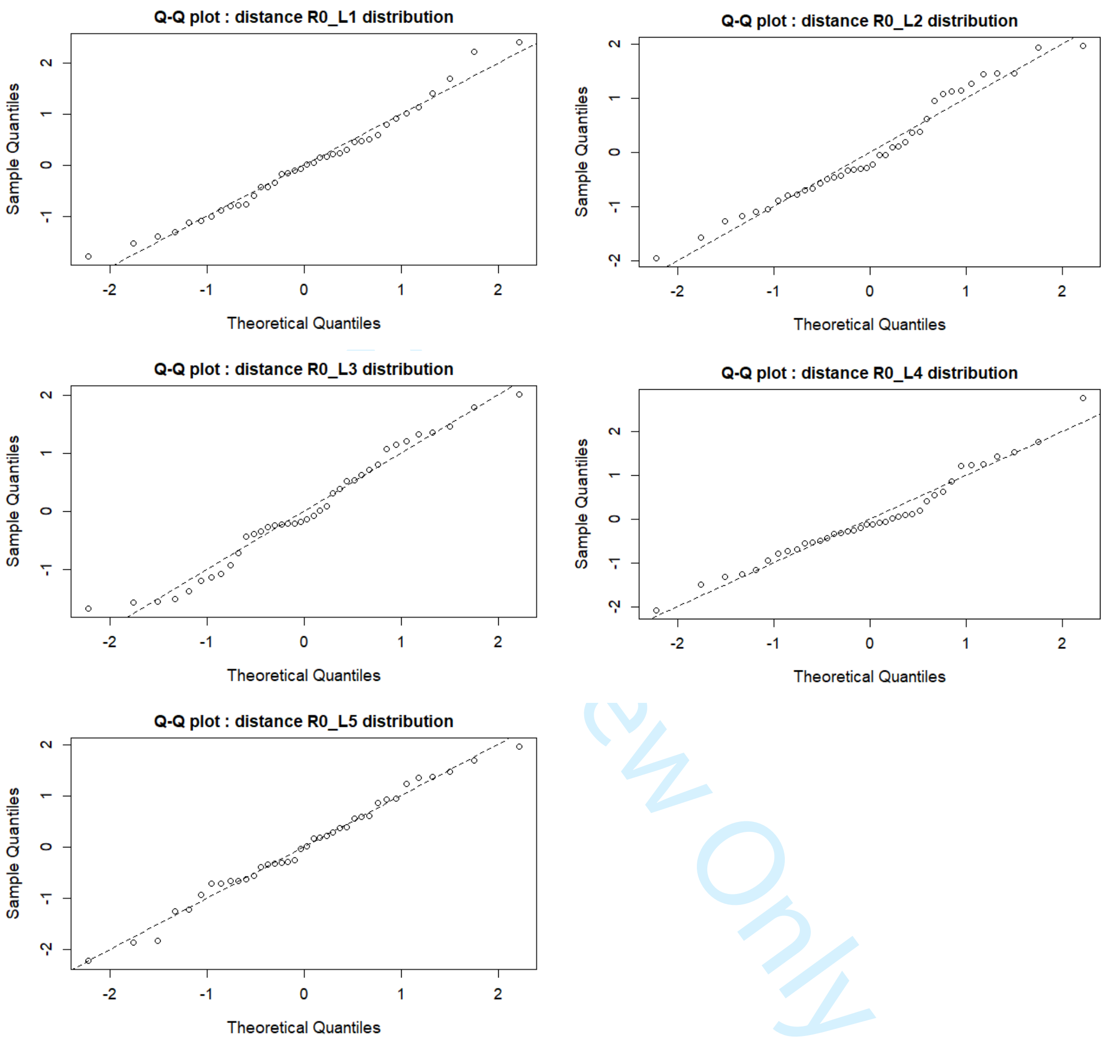

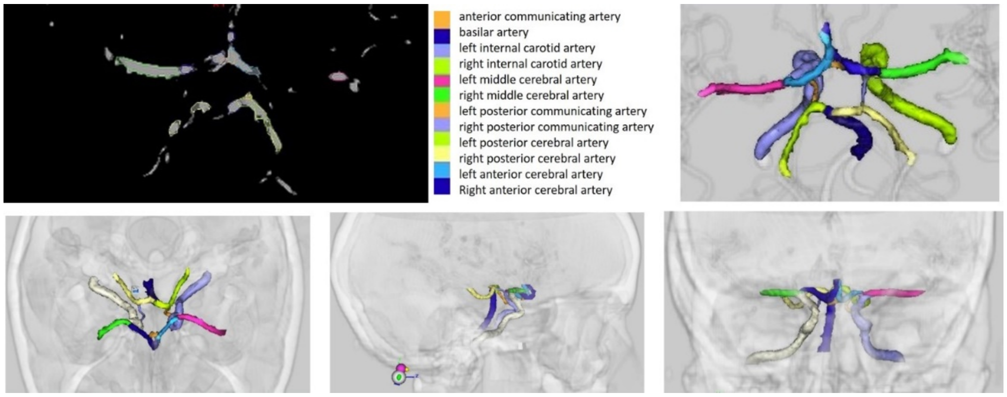

Figure 1- Contouring and labelling process.

133x52mm (220 x 220 DPI)

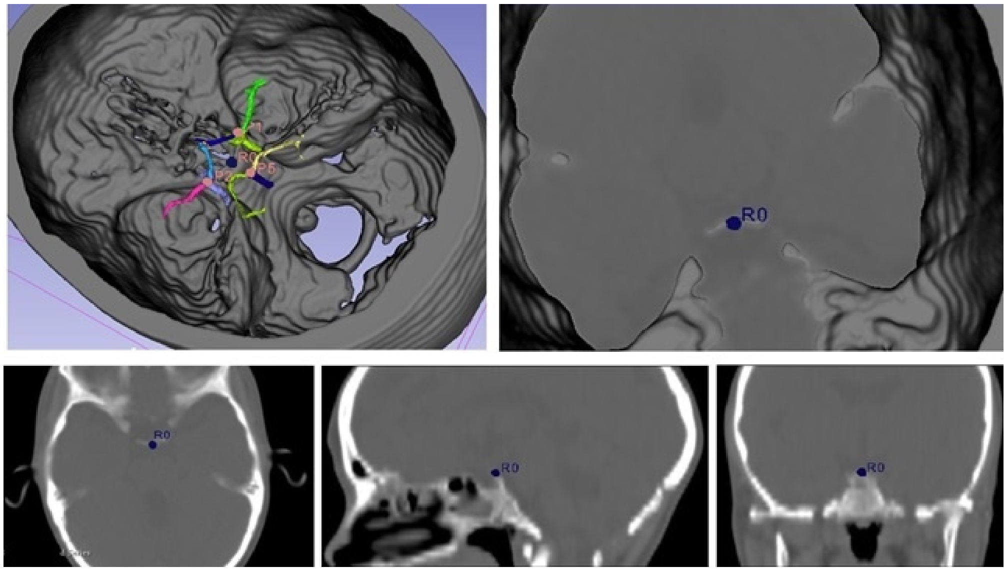

Figure 2- 3D representation of the segmented Willis Circle and the skull base.

74x41mm (220 x 220 DPI)

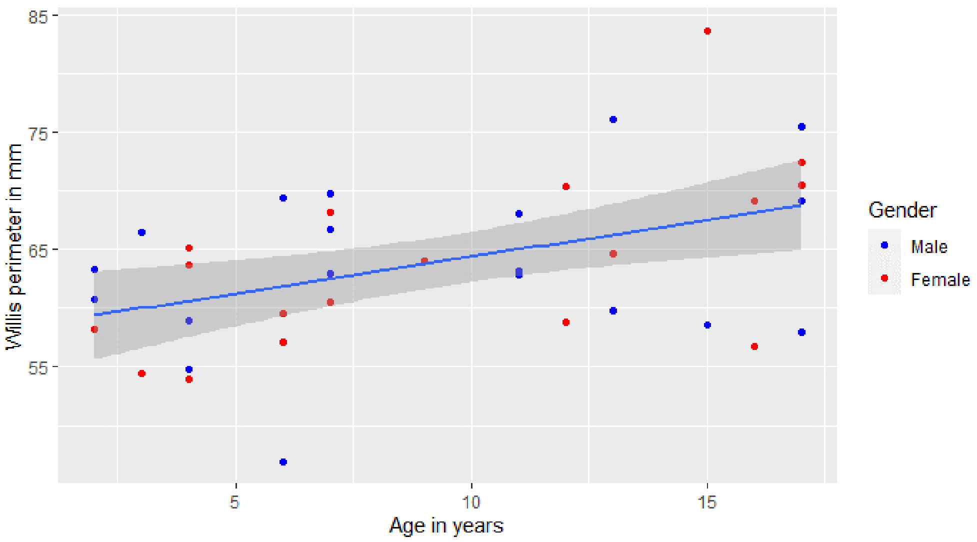

Figure 3- Willis perimeter growth according to age.

185x114mm (96 x 96 DPI)

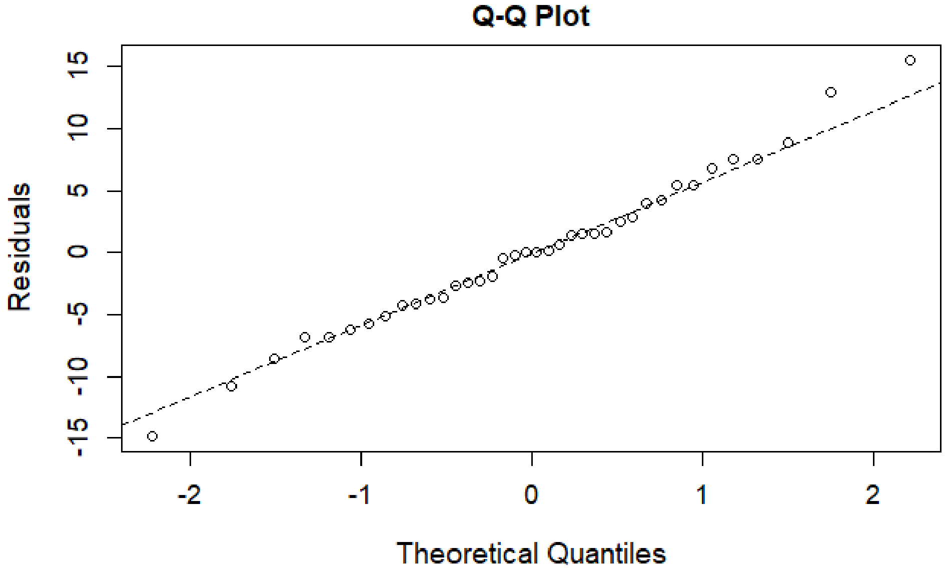

Figure 4- Residual L1 L2 L5 distances distribution.

173x107mm (96 x 96 DPI)
