# Supplementary material for: Topographic variability of the normal circle of Willis anatomy on a paediatric population
Source: Brain Commun. 2021 Apr 3;3(2):fcab055. doi: 10.1093/braincomms/fcab055 (PMC8204365; doi:10.1093/braincomms/fcab055)
Supplement: fcab055_Supplementary_Data [file fcab055_supplementary_data.zip › Review_files.zip!/Revision 1-Decision Letter.pdf]

Revision 1-Decision Letter

16-Feb-2021

Dear Dr. DIALLO,

It is a pleasure to accept your manuscript entitled "Topographic variability of the normal Circle of Willis anatomy on a pediatric population" for publication in Brain Communications. Any comments made by the reviewer(s) are included below, which can be addressed during the copy editing/proofs stage.

The next step is for your paper to go through “First Look”. We will contact you again shortly if any final files or formatting changes are required for publication or if we have any queries.

If no changes are needed, your submission will be transferred to our Production Department and you will hear from the Production Editor in due course.

Video abstracts - Brain Communications encourages authors to provide a video abstract to accompany their article. These are hosted on the Brain/Brain Communications YouTube channel. If you are interested in producing a 5-min video abstract to promote your article, then please contact the Editorial Office (brcoms.editorialoffice@oup.com) for further details and guidelines.

Thank you very much for your contribution. On behalf of the Editors of Brain Communications, we look forward to your future contributions to the Journal.

Yours sincerely,

Professor Richard Chin
Associate Editor
Brain Communications

Reviewer(s)' Comments to Author:

Reviewer: 1

Comments to the Author
The revisions made to the manuscript have improved it readability. Figure Legends have added context to the Figures. I am happy to support publication of this work.

Reviewer: 2

Comments to the Author
The authors have satisfactorily addressed my previous comments. However, there are a few typos that need addressing and the overall language in the paper needs to improve for easier read. Perhaps some of this would be addressed by journal staff whilst copy editing?
